# Hemoglobin is an oxygen-dependent glutathione buffer adapting the intracellular reduced glutathione levels to oxygen availability

**DOI:** 10.1016/j.redox.2022.102535

**Published:** 2022-11-16

**Authors:** Simone Fenk, Elizaveta V. Melnikova, Anastasia A. Anashkina, Yuri M. Poluektov, Pavel I. Zaripov, Vladimir A. Mitkevich, Yaroslav V. Tkachev, Lars Kaestner, Giampaolo Minetti, Heimo Mairbäurl, Jeroen S. Goede, Alexander A. Makarov, Irina Yu Petrushanko, Anna Bogdanova

**Affiliations:** a Red Blood Cell Research Group, Institute of Veterinary Physiology, and Center for Clinical Studies (ZKS), Vetsuisse Faculty, University of Zurich, Zurich, Switzerland; b Engelhardt Institute of Molecular Biology, Russian Academy of Sciences, Moscow, Russian Federation; c Theoretical Medicine and Biosciences and Experimental Physics, Dynamics of Fluids Group, Saarland University, Saarland and Homburg, Germany; d Department of Biology and Biotechnology “L Spallanzani”, Laboratories of Biochemistry, University of Pavia, Italy; e Medical Clinic VII, Sports Medicine, University Hospital Heidelberg, Heidelberg, Germany; f Department of Internal Medicine, Division of Oncology and Hematology, Cantonal Hospital Winterthur, Switzerland; g Zurich Center for Integrative Human Physiology (ZIHP), Switzerland

## Abstract

Fast changes in environmental oxygen availability translate into shifts in mitochondrial free radical production. An increase in intraerythrocytic reduced glutathione (GSH) during deoxygenation would support the detoxification of exogenous oxidants released into the circulation from hypoxic peripheral tissues. Although reported, the mechanism behind this acute oxygen-dependent regulation of GSH in red blood cells remains unknown.

This study explores the role of hemoglobin (Hb) in the oxygen-dependent modulation of GSH levels in red blood cells. We have demonstrated that a decrease in Hb O_2_ saturation to 50% or less observed in healthy humans while at high altitude, or in red blood cell suspensions results in rising of the intraerythrocytic GSH level that is proportional to the reduction in Hb O_2_ saturation. This effect was not caused by the stimulation of GSH *de novo* synthesis or its release during deglutathionylation of Hb's cysteines. Using isothermal titration calorimetry and *in silico* modeling, we observed the non-covalent binding of four molecules of GSH to oxy-Hb and the release of two of them upon deoxygenation. Localization of the GSH binding sites within the Hb molecule was identified. Oxygen-dependent binding of GSH to oxy-Hb and its release upon deoxygenation occurred reciprocally to the binding and release of 2,3-bisphosphoglycerate. Furthermore, noncovalent binding of GSH to Hb moderately increased Hb oxygen affinity. Taken together, our findings have identified an adaptive mechanism by which red blood cells may provide an advanced antioxidant defense to respond to oxidative challenges immediately upon deoxygenation.

## Highlights

• Hypoxia causes an increase in reduced glutathione (GSH) in red blood cells.
• Hemoglobin (Hb) reversibly non-covalently binds GSH.
• Out of four GSH molecules bound to oxy-Hb two are released upon deoxygenation.

## Graphical abstract

**Figure:**
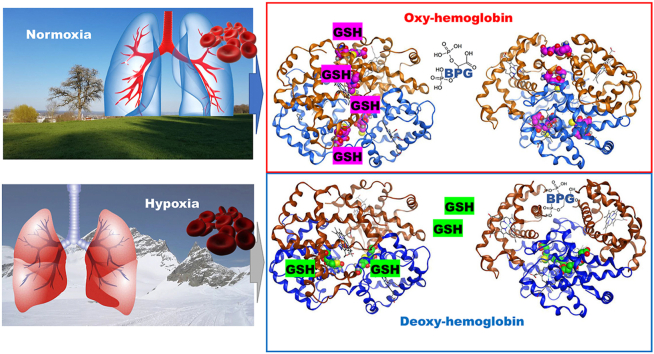

## 1 Introduction

The main function of the most abundant cells in our body, red blood cells (RBCs), is to mediate oxygen (O_2_) transport, and hemoglobin (Hb) is a key player in its execution. Apart from O_2_ delivery, RBCs with their robust antioxidative defense system scavenge H_2_O_2_ and function as a sink for oxidants produced by peripheral tissues under stress conditions, including hypoxia [1]. Millimolar concentrations of reduced glutathione (GSH) contribute substantially to the antioxidative defense system, along with superoxide dismutase, catalase, and peroxiredoxins [2,3]. GSH detoxifies oxidants by donating electrons while transforming into an oxidized state (GSSG). Glutathione also forms mixed disulfides with protein cysteines (S-glutathionylated adducts), protecting them from irreversible oxidation [4] and prolonging the lifespan of the circulating RBCs [2]. In addition, S-glutathionylation of regulatory thiols in Hb and transmembrane proteins, alters the cellular rheology, membrane stability, and O_2_-carrying properties of RBCs [[5], [6], [7]].

Changes in O_2_ availability in the micro- and macro-environment are associated with acute local or systemic alterations in the redox state. A transient bout of free radical production in the mitochondria of most cells is a part of the signaling cascade initiated in response to an acute hypoxic insult [8]. Prolonged continuous, or intermittent, exposure of humans to hypoxia is associated with systemic uncompensated oxidative stress that can be detected as depletion in blood levels of antioxidants [[9], [10], [11]]. The changes in GSH levels in RBCs upon prolonged hypoxic exposure depended on its severity and duration [[11], [12], [13]]. In order to compensate for hypoxia-induced oxidative stress, matching oxidative load and antioxidant defense capacity would be beneficial. Fast and flexible oxygen-dependent adjustments of the levels of antioxidants in RBCs are a part of this strategy. An acute dose-dependent increase in erythrocytic GSH levels in response to deoxygenation has been shown in mouse, rat, and human RBCs [[14], [15], [16]]. In rats a difference in free GSH levels were reported between the venous and arterial blood [14].

The mechanisms behind this tuning of GSH levels to O_2_ availability are currently unknown. Stimulation of the Embden Meyerhof pathway in response to deoxygenation is mediated by displacement of glycolytic enzymes from their docking sites on the cytosolic domain of band 3 protein by deoxyhemoglobin [[17], [18], [19]].This metabolic switch diverts glucose from pentose phosphate shunt to the ATP production required for *de novo* GSH synthesis [19,20]. However, an increase in GSH upon deoxygenation in mouse RBCs does not rely on *de novo* GSH synthesis [15]. The millimolar quantities of GSH emerging in the cytosol of deoxygenated RBCs exceed by far the amounts of GSH that may be recovered from the GSSG pool [14], but are comparable with the amount of GSH that may be liberated in the course of massive de-glutathionylation of the cysteine residues of Hb.

This study was undertaken to unravel the mechanisms of O_2_-dependent regulation of intraerythrocytic GSH in human RBCs. Herein, we demonstrate that Hb may function as an oxygen-sensitive buffer for GSH. Whereas oxyhemoglobin (oxy-Hb) non-covalently binds four GSH molecules, two of them may be released into the cytosol during the transition from oxygenated to deoxygenated Hb conformation.

## 2 Materials and methods

### 2.1 Blood samples and study participants

**Fig. 1:**
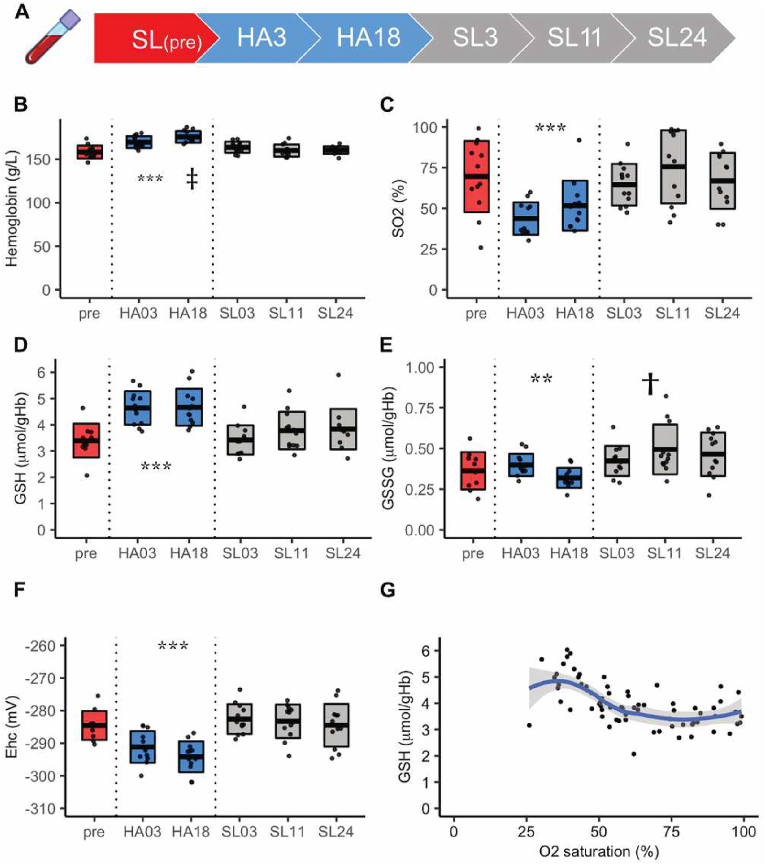
**The impact of high altitude on the selected blood parameters and intraerythrocytic redox state** (A) Study design with days of blood sampling. numbers in the arrows are the days of the study counted from ascent to the HA. Colors for the time of blood sampling (red for basal level, blue for HA exposure and grey for the time after descent from HA) are used in all the other plots. (B) Hemoglobin levels, and (C) SO_2_ measured by ABL825 FLEX, Radiometer. (D) intracellular GSH and (E) GSSG levels measured by Ellman's reagent. (F) Half-cell redox potential (E_hc_) for GSH/GSSG couple. Details may be found in Materials and methods section and in Ref. [21] (G) Association between SO_2_ and GSH levels. Blue line is a fit using the method of loess (Local Polynomial Regression Fitting), the grey area shows the confidence interval (95%). Statistics: N = 12, ANOVA with custom contrast: * HA different from pre/sea level; ‡ different HA03 and HA18; † different pre and sea level. *HA: High altitude (3500 m), SL: sea level (110 m), GSH: reduced glutathione, GSSG: oxidized glutathione, SO*_*2*_*: hemoglobin oxygen saturation.* (For interpretation of the references to colour in this figure legend, the reader is referred to the Web version of this article.)

The study at high altitude was approved by the ethics committees of the University of Heidelberg, Germany, (S-066/2018) and the University of Bern, Switzerland (2018-01766). Twelve young male subjects took part in the study and blood samples were collected at sea level (SL) and high altitude (HA) (see Fig. 1A and [22]).

In addition, nine venous blood samples of anonymized healthy donors of both genders were provided by the Clinical Laboratory of Cantonal Hospital Winterthur, Switzerland. These samples were used for routine calibration of blood analyzers (CO-oximetry included). In agreement with the Helsinki convention, healthy study subjects gave informed consent for blood donation. Li-heparin was used as anticoagulant.

Deidentified study participants’ data that underly the reported results will be made available 3 months after the publication for a period of 5 years after the publication date at https://zenodo.org/.

### 2.2 Free intracellular GSH and GSSG measurement using Ellman's reagent

GSH measurements were performed immediately after opening of the vacutainers with blood samples or harvesting the samples from Eschweiler tonometers, in parallel to CO-oximetry. The samples were deproteinized by mixing with 5% trichloroacetic acid. Ellman's reagent was used to determine the levels of GSH and oxidized glutathione (GSSG) in protein-free supernatant obtained after sedimentation of protein pellet as described elsewhere [15,23]. Non-protein reduced thiol levels detected using Ellman's reagent are mainly presented by GSH in RBCs as free cysteine levels are negligibly low (about 5 μM are reported in human RBCs [24]). Enzymatic reduction of GSSG to GSH using glutathione reductase allows quantitative and specific detection of GSSG [23]. Half-cell redox potential for GSH/GSSG couple was calculated from the equation

$$E_{hc}\left(mV\right)=-240-\left(\frac{59.1}{2}\right)\times \log\frac{{\left[GSH\right]}^{2}}{\left[GSSG\right]}$$

at 25 °C, pH 7.0, as described elsewhere [21].

### 2.3 Effect of hypoxic exposure on the intraerythrocytic GSH *ex vivo*

Oxygen-dependence of GSH levels in RBCs: RBC were washed in bicarbonate-containing plasma-like buffer BPLB (125 mM NaCl, 25 mM NaHCO_3_, 4 mM KCl, 10 mM glucose, 300 μM l-Arginine, 300 μM glutamic acid monosodium, 300 μM glycine 10 mM HEPES-imidazole (pH = 7.40 at room temperature) and subsequently resuspended in BPLB and transferred into the Eschweiler tonometers. Buthionine sulfoximine (BSO, 1 mM) was added to specific blood samples to inhibit *de novo* GSH synthesis. Blood samples were pre-equilibrated with gas phase containing 15% O_2_ for 30 min and then O_2_ level was reduced to 0.5% O_2_ and incubation continued for further 2 h. Aliquots were collected at various time points to assess Hb O_2_ saturation (SO_2_) with ABL825 FLEX blood analyzer and intracellular GSH and GSSG as stated above.

The impact of N-ethyl maleimide (NEM) on GSH release under hypoxic conditions: RBC were washed and resuspended in the plasma-like buffer (PLB). Incubation with NEM (20 mM, 20 min) was used to derivatize reduced thiol groups and the excess of it was removed by triple-washing. RBC suspensions were subsequently placed into the Eschweiler tonometers and equilibrated with the air or pure N_2_ as gas phase at 37 °C, and intracellular GSH levels and SO_2_ were measured thereafter.

GSH levels in hemolysates treated with BPG: Washed RBC suspensions of 20% hematocrit (6.7 g/dL Hb, ∼1 mM) were resuspended in PLB and lysed by snap-freezing-thawing. Two lysate samples were placed into Eschweiler tonometers and exposed to oxygenation-deoxygenation-reoxygenation protocol (each step lasting 15 min). One of the samples was supplemented with 2 mM 2,3-bisphospho-d-glycerate (BPG) while deoxygenated-reoxygenated. The Hb:BPG ratio made up 1:2. Hb oxygen saturation SO_2_ and cytosolic free GSH were detected in lysates at each condition.

### 2.4 Flow cytometry

For *ex vivo* deoxygenation experiments suspensions of RBC in BPLB were exposed to hypoxia (1% O_2_) or normoxia (20% O_2_) at 37 °C for 3 h using Whitley H45 HEPA Hypoxystation or cell culture CO_2_ incubator respectively. Half an hour prior to the end of hypoxic exposure 1 μl RBC suspension was mixed with 99 μl of the BPLB equilibrated with the gas phase of the chamber or CO_2_ incubator and RBCs were then loaded with fluorescent dyes to detect reduced thiols, reactive oxygen species, or NO for 30 min at 37 °C in the same conditions (hypoxia or normoxia) in the dark [25,26]. Bulk intracellular reduced thiols were measured using monobromobimane (mBBr, 20 μM), ROS assessed using dihydrorhodamine 123 (DHR, 7.5 μM) and N_2_O_3_ as a marker of NO was measured using 4,5-Diaminofluorescein diacetate (DAF-FM DA, 5 μM). After loading with the dyes, suspensions were diluted with BPLB pre-equilibrated under hypoxic or normoxic condition and fluorescence was recorded using BD LSR Fortessa Flow Cytometer.

Samples from HA study were collected for detection of reduced bulk (non-protein and protein) thiols by flow cytometry. Whole blood sample was loaded with 10 μM mBBr, in plasma-like buffer supplemented with 0.1% bovine serum albumin for 1h in the darkness at room temperature [26] and RBC fluorescence was recorded using Gallios Flow Cytometer, BC.

### 2.5 Quantification of free GSH content in RBCs by ^1^H NMR nuclear magnetic resonance spectroscopy

After incubation of the RBC suspensions in normoxia or hypoxia a 5% solution of trichloroacetic acid in D_2_O was added to them. Precipitated protein was pelleted by centrifugation and the supernatants were placed on ice. An aliquot (410 or 415 μL) of supernatant was placed into standard 5 mm NMR tube. Standard solution of 0.5 μg/μL GSH in D_2_O was prepared afresh before each experiment. Series of spectra was recorded concomitantly with sequential addition of GSH standard solution aliquots directly into NMR tube using thin glass capillary. Volume of each aliquot was determined gravimetrically. For each spectral series, the signal of cysteine β-protons (at 2.9 ppm) characteristic for GSH, was integrated after the phase and baseline correction. The initial content of GSH in each sample was then determined from the integral intensities, by linear regression (Excel), as well as the standard error.

^1^H NMR spectra were recorded using 300 MHz Avance 3 spectrometer (Bruker), at temperature of 303K, using Watergate W5 pulse sequence [27] for water signal suppression. Each spectrum was accumulated from 192 scans.

### 2.6 Isothermal titration calorimetry (ITC)

Purified Hb solution was prepared as follows. Human met-Hb (0.1 mM) was dissolved in 50 mM K-phosphate buffer (50 mM KCl, 34.8 mМ K_2_HPO_4_, 15.2 mM KH_2_PO_4_, 2 mM MgCl_2_, pH 7.4) and reduced using 5 mM sodium dithionite for 10 min. Reduced Hb was passed through the gel-filtration column (PD MiniTrap G-25). Elution was performed using 50 mM K-phosphate buffer. GSH (4 mM) and BPG (1 mM) were allowed to interact with oxy- or deoxy-Hb or Hb-GSH complex.

Experiments were performed in the normoxic atmosphere (air), or under hypoxic conditions (1% O_2_). To achieve hypoxia the ITC device system was placed into a hypoxic chamber (Whitley H45 HEPA Hypoxystation) to reach stable deoxygenating conditions (99% N_2_, and 1% O_2_) (Fig. S1). Prior to the experiment under hypoxic conditions all the buffers and the MiniTrap column were equilibrated with the atmosphere of the hypoxic chamber overnight. All manipulations with Hb during its preparation were also performed in hypoxic atmosphere. All stock solutions were prepared on the buffer pre-equilibrated in hypoxic atmosphere of the Hypoxystation. Experiments were carried out at 25 °C in a 50 mM K-phosphate buffer. Aliquots of the ligand (2.0 μl, GSH 4 mM or BPG 1 mM) were injected into the cell containing 70–100 μM Hb to obtain a complete binding isotherm. To detect the effective heat of binding, the heat of dilution was subtracted from the heat of the reaction.

The thermodynamic parameters for the GSH and BPG binding to Hb were determined using a MicroCal iTC200 and PEAQ-ITC, as described elsewhere (23).

The resulting titration curves were fitted using the MicroCal Origin and Malvern software, assuming one set of binding sites. Equilibrium association constant (*K*_*a*_) (dissociation constant *K*_*d*_ = 1/*K*_*a*_), the enthalpy change (ΔH) and stoichiometry of binding (N) were determined, and the changes in Gibbs energy (ΔG), and the entropy change (ΔS) were calculated from equation ΔG = -RT lnKa = ΔH-TΔS.

### 2.7 Hemoglobin oxygen affinity measurement

Hb oxygen affinity was measured in hemolysates prepared by freezing-thawing of RBC suspensions (Hct of 30%) in PLB. The 20 μl aliquots of hemolysates were added to 5 ml PLB without or with 5 mM GSH and O_2_ dissociation curves using Hemox analyzer (TCS Scientific, New Hope, PA).

### 2.8 Analysis of solvent-accessible surface area in Hb structures

Structures of oxygenated (5WOG; 5WOH and 1HHO), deoxygenated (1A3N; 2HHB and 2DN2) and of hemoglobin-BPG complex (1B86) were obtained from protein data bank (rcsb.org). Solvent accessible surface area (SASA) was calculated using Accessible Surface Area and Accessibility Calculation for Protein server and GETAREA server using a spherical water probe with a fixed radius (1.4 Å). Analysis of the cysteine S-groups positioning was performed using Moe2015.10 software.

### 2.9 Docking of GSH to Hb and Hb-BPG complex

For docking of GSH to Hb structures of human oxy-Hb and deoxy-Hb were obtained from the protein data bank (rcsb.org). For docking of GSH to Hb-BPG complex we have used the structure of human Deoxy-Hb–BPG complex (1B86) from the protein data bank (rcsb.org). Structures were pre-processed using the AutoDockTools program. Docking was performed using Autodock Vina. Moe2015.10 software was used for analysis of receptor-ligand interactions.

### 2.10 Investigation of the possible impact of deoxygenation on the Hb S-glutathionylation state

Blood samples collected from 12 study participants at sea level and high altitude (see Fig. 1A and [22] for more details). Washed RBC pellet was snap-frozen in liquid nitrogen and stored at −80 °C. Samples were thawed on ice and supplemented with a protease inhibitor cocktail (1:200) and 25 mM NEM in PBS to prevent oxidation of SH-groups. Negative control was prepared by treatment of a sample with 100 mM dithiothreitol. Primary mouse Anti-Glutathione antibody (ab19534, abcam 1:1000) were used for detection of S-glutathionylated proteins and rabbit anti-human Recombinant Anti-Hemoglobin subunit beta/ba1 antibody (ab214049, Abcam 1:1000) for detection of Hb. Densitometry was performed using Image studio lite (LI-COR Biosciences) and the signal for GSH was normalized to that for the βHb monomer. Finally, the readouts obtained for different time points were normalized to the basal level of glutathionylation (time-zero for *ex vivo* experiments or samples before the HA exposure). Similar procedure was performed with RBC hemolysates obtained from RBC suspensions exposed to air or pure N_2_ or air in Eschweiler tonometers at 37 °C.

### 2.11 Statistics

Statistical analysis was performed using the R software [28] and GraphPad Prism. Shapiro-Wilk test was used for checking if the data were normally distributed. Subsequently, a parametric paired Student's t-test or non-parametric Wilcoxon test was used to compare the two experimental groups. Specific details can be found in figure legends. Values are shown either as means ± standard deviations or as boxplots with the minimum, the maximum, the sample median, and the first and third quartiles. To investigate the effects of the repeated measures in the *in vivo* HA study, an analysis of variance (ANOVA) was performed with the study participant as a random factor. The differences between the 7 days were decomposed into four contrasts and tested on the level “day x study participant”. The first contrast tested whether the dependent variable was different at high altitude compared to sea level (*p value < 0.05, **p value < 0.01, ***p value < 0.001). Then, the second contrast tested the difference between the two time points at high altitude (‡p value < 0.05, ‡‡p value < 0.01, ‡‡‡p value < 0.001). Third, “pre” levels were compared to levels after return to sea level (†p value < 0.05, ††p value < 0.01, †††p value < 0.001) and the last contrast tested whether the days after return to sea level were different (¥).

## 3 Results

The effect of three weeks-long exposure to hypobaric hypoxia experienced at high altitude (HA) on RBC turnover in twelve healthy male students from Heidelberg was explored at Jungfraujoch research station in Switzerland (3500 m above sea level (SL)) as described by Klein et al. [22] and schematically shown in Fig. 1A. Exposure to HA was associated with an increase in Hb levels (Fig. 1B) and a decrease in Hb O_2_ saturation (SO_2_) of venous blood (Fig. 1C) compared to the values at SL. At the same time, intracellular GSH levels in RBCs increased from 3.4 ± 0.65 to 4.6 ± 0.64 μmol/g Hb (p < 0.05), when at HA, and immediately returned to basal levels when the study participants returned to SL (Fig. 1D). These changes were not accompanied by the reciprocal changes in GSSG of a corresponding size (within 0.5 μmol/g Hb range) (Fig. 1E). As a result, a reduction in the intraerythrocytic half-cell redox potential (E_hc_) was observed under conditions of hypobaric hypoxia. Descent back to the SL was associated with recovery of E_hc_ back to the basal levels (Fig. 1F). Plotting the data for intraerythrocytic GSH as a function of SO_2_ revealed that dose-dependent exponential growth of intracellular GSH was only observed at a SO_2_ below 50% (Fig. 1G).

**Fig. 2:**
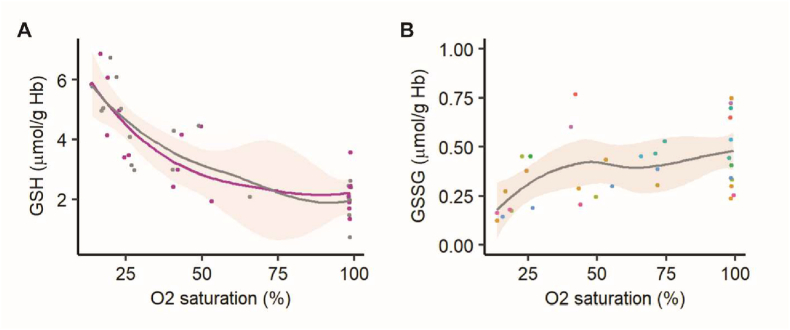
**Recycling from GSSG and de novo synthesis do not explain the increase in GSH levels upon deoxygenation**. (A) Intraerythrocytic GSH levels as a function of SO_2_ in RBC suspension (N = 7). Hemoglobin was pre-equilibrated with 15% O_2_ for 30 min and then deoxygenated gradually by switching to 0.5% O_2_ for 2 h. Grey curve is for untreated control, and pink curve is for RBC pretreated for 20 min with the inhibitor of *de novo* GSH synthesis BSO (l-Buthionine-sulfoximine, 1 mM). (B) GSSG levels in RBCs as a function of SO_2_ (N = 9). *Abbreviations identical to*Fig. 1. (For interpretation of the references to colour in this figure legend, the reader is referred to the Web version of this article.)

In line with these findings for the *in vivo* hypoxic exposure, gradual deoxygenation of RBCs resuspended in a plasma-like buffer using a gas mixture containing 0.5% O_2_, 5% CO_2_, and 94.5% N_2_ resulted in a similar increase in intracellular GSH that was initiated when SO_2_ reached 60-50% and progressed with further deoxygenation (Fig. 2A, grey line). Pre-treatment of RBCs with an inhibitor of *de novo* GSH synthesis BSO did not prevent this hypoxia-induced rise in intracellular GSH (Fig. 2A, purple line). Both *in vivo* and *in vitro* deoxygenation of RBCs was not associated with a decrease in GSSG levels which could serve as a source of the GSH increase (Fig. 2B).

**Fig. 3:**
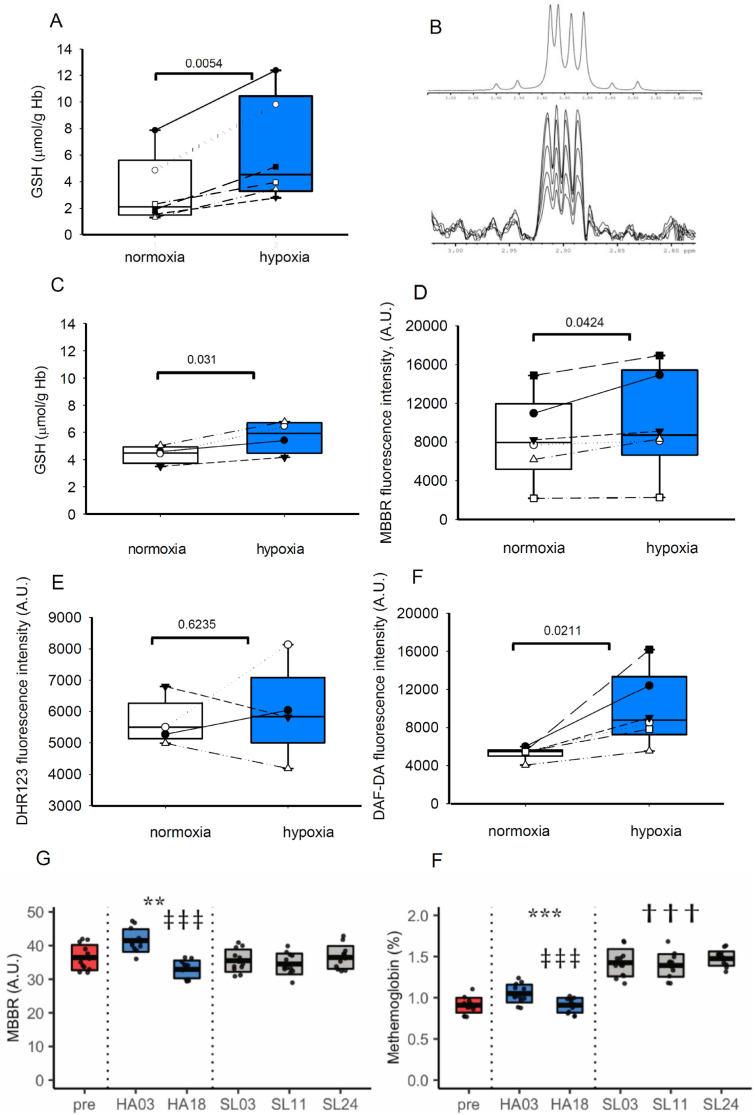
**The impact of deoxygenation on redox balance in RBCs ex vivo and in vivo.** The effect of incubation of RBC suspensions at 1% O_2_ (hypoxia) for 3h on: (A) GSH levels measured by Ellman's reagent (N = 6, paired Student's t-test) (B) GSH quantification by ^1^H NMR. Region of reference ^1^H spectrum for the standard GSH solution corresponding to Cys β-protons (upper panel), and in the same region in spectra of deproteinized RBC samples obtained by consecutive addition of standard GSH solution aliquots (lower panel). Both proton signals are of dd shape (14.2Hz and 5.4Hz; 14.2Hz and 6.7 Hz), but, as the spectrum is not one of the first order, only the central components comprising the most of signal intensity (range between 2.93 and 2.87 ppm) were used for integration, to minimize the contribution of noisy baseline. (C) Quantification of GSH in the samples obtained from oxygenated and deoxygenated RBC by means of ^1^H NMR normalized per Hb levels (N = 4, paired Student's t-test) (D) Bulk reduced thiols detected using MBBR staining by means of flow cytometry (N = 6, paired Student's t-test). (E) ROS levels detected using DHR123 (N = 5 paired Student's t-test) and (F) N_2_O_3_ levels measured using DAF-DA (N = 6, paired Student's t-test). (G) Bulk thiol levels detected using MBBR fluorescence recorded by flow cytometry, and (H) metHb measured using ABL825 FLEX, Radiometer in RBCs of 12 participants of the high altitude exposures study For more details on the HA study design see Fig1A. N = 12, ANOVA with custom contrast: * HA different from pre/sea level; ‡ different HA03 and HA18; † different pre and sea level. *MBBR: monobrombimane, NO: nitric oxide, ROS: reactive oxygen species, DHR: dihydrorhodamine, DAF-DA: diaminofluoresceine diacetate.*

As Ellman's method detects all non-protein reduced thiols and is not entirely specific for GSH the observations were verified using two independent experimental techniques, Ellman's method (Fig. 3A) and ^1^H NMR spectroscopy (Fig. 3C). Sample preparation was identical for both sets of measurements. Deoxygenation of RBC suspensions was performed in a hypoxic chamber at 1% O_2_. NMR spectroscopy enabled us to avoid possible interference from any constituents of cellular extracts containing reduced thiols other than GSH. This was possible because the measured quantity reflected the amount of reduced glutathione *Cys* β-protons giving rise to specific signal at 2.90 ppm (Fig. 3B, upper panel). The absolute quantities of GSH in the samples were determined by titration of the samples with pure GSH solution of known concentration (Fig. 3B, upper panel). Individual experimental readouts are shown in Figs. S2A–D. The obtained values for GSH levels in the samples were normalized to Hemoglobin content in them (Fig. 3C). Comparison of the data in Fig. 3A (for the Ellman's assay) and **3C** (for NMR spectroscopy) revealed that hypoxic exposure was indeed inducing an increase in GSH levels. One more methodological approach complementary to that using Ellman's method was used to test if hypoxia has impact on the bulk (protein and non-protein) reduced thiol levels in RBCs. Fluorescence of monobromobimane adducts with reduced bulk thiols was used as a readout. As follows from Fig. 3D, acute deoxygenation of RBC suspension increased the abundance of bulk reduced thiols.

The observed accumulation of GSH in the cytosol of hypoxic RBCs in suspension was not associated with the changes in reactive oxygen species (ROS) production (Fig. 3E) but with the rise in the intracellular N_2_O_3_ as a marker of NO levels (Fig. 3F).

Systemic exposure of our study participants to HA hypoxia for 3 days was associated with an increase in both bulk thiols in RBCs (Fig. 3G) as well as intraerythrocytic GSH levels (Fig. 1D) However, after 3-week-long stay at HA bulk (protein and non-protein) thiols were decreased compared to the basal level (pre values), whereas GSH levels in RBCs remained elevated (compare Fig. 1D and Fig. 3FG). Thus, long-term hypoxia supported oxidation of protein thiols. Methemoglobin levels remained unchanged while at HA but increased after descent from the HA back to the sea level and the concomitant reoxygenation (Fig. 3GF).

**Fig. 4:**
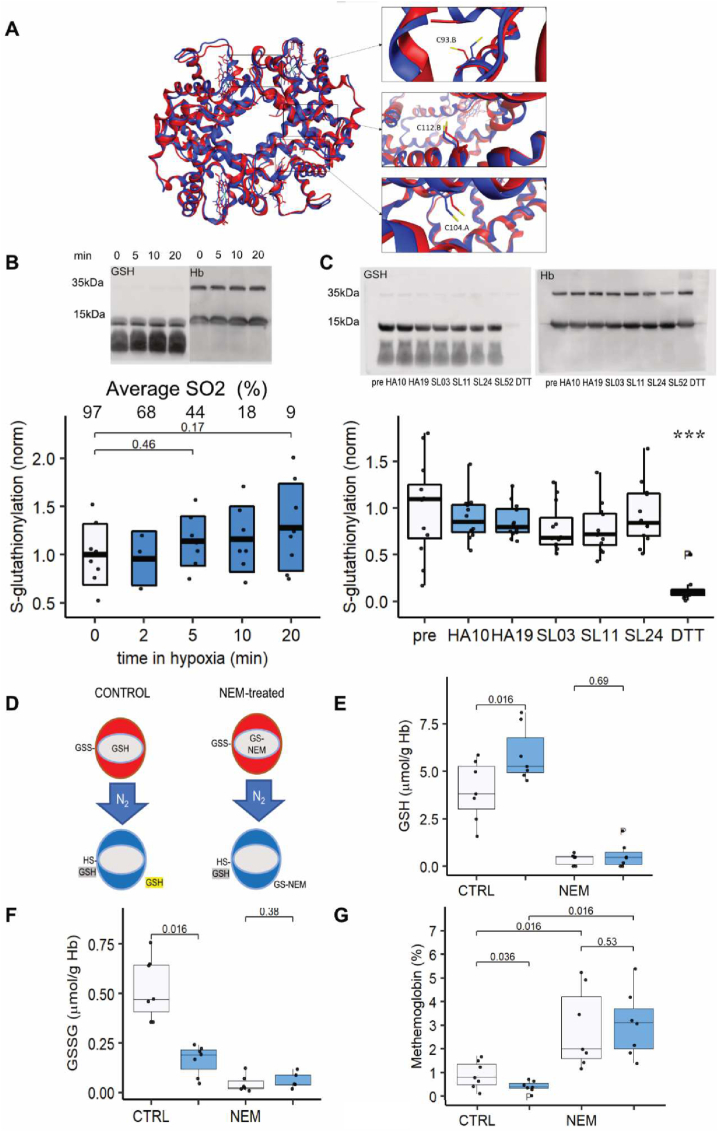
**Discrimination between oxygen-dependent covalent interaction of GSH with Hb (Hb S-glutathionylation) and non-covalent docking of GSH to Hb: (**A) In silico modeling of the possible changes in availability of Cys residues αCys104, βCys93, and βCys112 for S-glutathionylation. Shown is a superposition of oxy-Hb(blue) and deoxy-Hb(red) structures with a blow-up of the areas where Cys are localized. While thiol group of βCys93 is facing outwards in the deoxy-Hb, it is turned inwards into the Hb molecule in the oxy-Hb state. Position of the thiol groups of αCys104 and βCys112 is not altered by oxygenation-deoxygenation. (B) S-glutathionylation of Hb in RBC suspension that were gradually deoxygenated in a tonometer in the atmosphere of 100% N_2_. Aliquots were taken after 2, 5, 10, and 20 min and SO_2_ shown above the plot was measured by ABL825 FLEX, Radiometer. RBC were lysed in non-reducing lysis buffer supplemented with NEM (25 mM) and S-glutathionylation of Hb detected using specific antibodies and normalized to the signal for β-globin as a loading control. Representative blots obtained for one of the RBC samples is shown at the upper panel. Densitometry for the S-glutathionylated form of Hb monomers normalized to that for the β-globin is shown at the lower panel. Paired Wilcoxon test was used for analysis. N = 8. (C) Similar approach was used to assess S-glutathionylation of Hb in blood samples of participants from the HA study. Representative immunoblot is shown for one participant before, during and after the staying at HA. Statistics: ANOVA with custom contrast * different from pre/sea level and high altitude. N = 12 (D) Schematic representation of NEM experiment. GSH levels were detected in control untreated oxygenated RBCs that were sham-washed and those oxygenated cells that were pre-treated with 20 mM NEM for 20 min at 21% O_2_ and the washed free from the NEM that did not interact with the targets (red circles). Thereafter, both NEM-treated and control non-treated RBCs were deoxygenated with 100% N_2_ for 30 min (blue circles) and GSH detected once again. Further explanations to the scheme may be found in the text. GSH (E) and GSSG (F) were detected using Ellmann's reagent and metHb levels (G) was measured by ABL825 FLEX, Radiometer. Paired Wilcoxon test. N = 7. *NEM: N-ethyl malemide. RBC: red blood cell, DTT: 1,4-Dithiothreitol, HBB: Hemoglobin subunit beta, GSH: reduced and GSSG: oxidized Glutathione.* (For interpretation of the references to colour in this figure legend, the reader is referred to the Web version of this article.)

We hypothesized that Hb is capable to covalently or non-covalently interact with GSH, and that conformation transition from oxy-Hb to deoxy-Hb may result in release of GSH from its binding site(s). Availability of cysteine residues α-104, β-112, and β-93 for S-glutathionylation was modelled *in silico* for the oxy- and deoxy-Hb. Analysis of the available crystal structures of oxy- and deoxy-Hb reveals that the thiol group of the βCys93 is the only thiol inwardly facing in oxy-Hb but translocates into an outwardly facing position as Hb deoxygenates (Fig. 4A) while the other thiols are not displacing with the changes in oxygenation. If GSH is bound to this thiol group, its de-glutathionylation is only possible when Hb is in deoxygenated state and glutaredoxin 1 may approach it (Table S1). The thiol group of the αCys104 is unavailable for S-glutathionylation and that βCys112 is available to bind or release GSH independent of Hb oxygenation state follows from the analysis of solvent accessible surface area (Table S1).

To verify if the changes in Hb oxygenation are associated with an alteration to the S-glutathionylation of Hb molecules (HbSSG), RBCs were exposed to normoxic or hypoxic atmosphere, Hb harvested, and immunoblotting was performed using the antibodies against glutathione and the alpha-chain of Hb (Fig. 4B). A similar approach was used to compare HbSSG abundance in the blood of study participants after 3 weeks at HA and when at SL (Fig. 4C). RBCs treated with dithiothreitol were used as a negative control. The representative images show the alpha-globin monomers (∼15–17 kDa) and the dimers containing the alpha-globin (∼32–35 kDa). S-glutathionylation of the monomers is clearly observed. However, the samples obtained from RBCs exposed to acute (minutes) *in vitro* or long-term *in vivo* (days) hypoxia show no changes in S-glutathionylated adducts when compared to the corresponding normoxic samples (Fig. 4B&C).

To exclude the possibility of de-glutathionylation as a source of GSH release at low O_2_ tension, we used N-ethylmaleimide (NEM) to block SH-groups of GSH and proteins from interaction with each other. The experiment is schematically described in Fig. 4D. The cells were pre-treated with NEM while oxygenated, and the excess of NEM that did not react with thiols was washed away. The aliquots of normoxic control and NEM-treated RBC suspensions were harvested and the depletion of non-protein thiols confirmed (Fig. 4E, open bars). Thereafter the control and NEM-pretreated suspensions were deoxygenated and another measurement of cytosolic free GSH was performed (blue bars in Fig. 4E). In control samples that were not pre-treated with NEM an increase in GSH was observed upon deoxygenation as expected. In NEM-treated samples no liberation of GSH from the stores was observed. GSSG pool was somewhat reduced after deoxygenation in control samples (not enough to cover for the increase in GSH) and did not change in NEM-treated samples (Fig. 4F). Treatment with NEM resulted in accumulation of met-Hb, but this accumulation was independent of the oxygenation of Hb (Fig. 4G). The outcome of this experiment supported the non-covalent nature of docking of GSH to Hb as NEM could reach out for the reduced thiol group of GSH in oxygenated RBCs (the GSH molecule schematically showing enclosed into Hb in the scheme in Fig. 4D. If the source of GSH liberated from Hb during deoxygenation would be S-glutathionylated adducts, they would not be available for NEM to form conjugates, and GSH release after deoxygenation (the GSH highlighted in yellow in Fig. 4D) would still occur in NEM-pretreated samples.

**Fig. 5:**
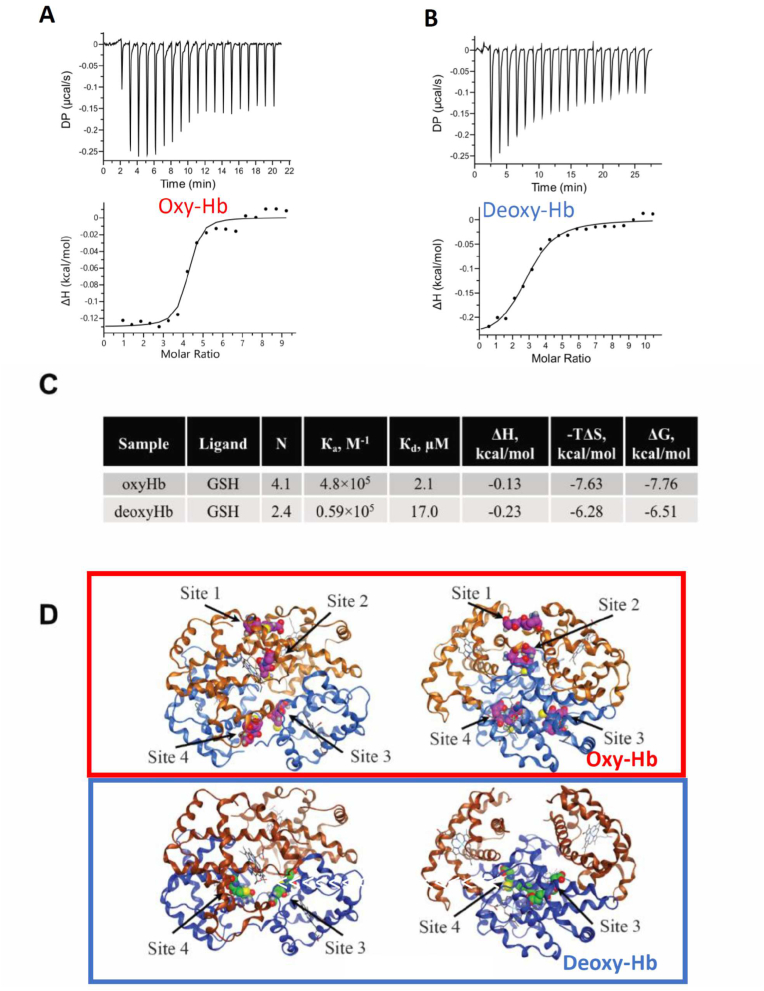
**Noncovalent binding of GSH to Hb is O**_**2**_**-dependent**: ITC titration curve (upper panel) and binding isotherm (lower panel) for GSH interaction with (A) oxy-Hb (21% O_2_) and at (B) deoxy-Hb (1% O_2_) at 25 °C. (C) Thermodynamic parameters of GSH binding to oxy-Hb and deoxy-Hb determined by isothermal titration calorimetry. Shown are the number of binding sites for GSH per 1 molecule Hb (N), and association and dissociation equilibrium constants K_a_ and *K*_d_, and the thermodynamic parameters of Hb:GSH interaction ΔH, -TΔS and ΔG. (D) Best Hb:GSH docking models produced by Vina Autodock docking. Upper panel shows the front and side views for the best four affinity GSH binding sites in the oxy-Hb, and lower panel shows the same for the deoxy-Hb. Beta chains are shown in brown, and the alpha chains are in blue. Shown in pink are GSH molecules occupying the sites 1–4 and in oxy-Hb. The GSH molecules in green interact with the sites 3 and 4 in deoxy-Hb. *ΔH: the changes in enthalpy, -TΔS: the changes in entropy, and ΔG: the changes in Gibbs energy. ITC: isothermal titration calorimetry. (For interpretation of the references to colour in this figure legend, the reader is referred to the Web version of this article.)*

In the next set of experiments we have monitored the non-covalent binding of GSH to purified oxy-Hb and deoxy-Hb protein using isothermal titration calorimetry (Fig. 5A and B). By using Hb with reduced thiol groups and a reduced form of glutathione in the experiment, we could exclude from consideration the possibility of forming a covalent S–S bond between Hb and GSH. The spontaneous S-glutathionylation reaction that does not require a catalyst occurs only as a result of dithiol exchange between the protein thiol and the GSSG. Binding of GSH to oxy-Hb and deoxy-Hb was detected, as shown in the original ITC titration curves (Fig. 5A and **B** respectively). The thermodynamic parameters such as the changes in enthalpy (ΔH), in entropy (TΔS), and in Gibbs energy (ΔG), equilibrium association and dissociation constants K_а_ and *K*_d_, and stoichiometry (N) of interactions were obtained in these experiments (Fig. 5C). Oxy-Hb was shown to bind four molecules of GSH, whereas deoxy-Hb could only bind two of them. Affinity of deoxy-Hb to GSH (*K*_d_ = 17 μM) was lower than that of the oxy-Hb (*K*_d_ = 2 μM) and the entropic component prevailed in the binding energy profile (Fig. 5C). This indicates that the binding can be attributed primarily to hydrophobic interactions between the GSH and the protein.

*In silico* modelling was used to identify the amino acid residues involved in non-covalent docking of GSH to Hb (Table S2 and Fig. 5D). In oxy-Hb, two pairs of “pockets” were found, each binding a single GSH molecule. The first pair of binding sites is located at the interface between the two β-globins, and the second pair of sites with slightly lower affinity is located between the two α-globins, both pairs of sites spaced within the central cavity (Fig. 5D, upper panel).

Amino acids predicted to be involved in GSH interaction with Hb are listed in Supplementary Table S2. The following key conservative amino acids were predicted to form each of four sites shown in Fig. 5D (site 1: β_1_Val1, β_1_Leu81, β_1_Ala140, β_1_His146, and β_2_Leu81, β_2_Ala140, β_2_His146; site 2: β_1_His146 and β_2_Ala138, β_2_His146; symmetrical sites 3 and 4: α_1_Val1, α_1_Leu2, α_1_Pro95, α_1_Phe98, α_1_His103, α_1_Asp126, α_2_Tyr 140, β_1_Val 34, β_1_Trp37, β_1_Asn108, β_1_Val109). Upon deoxygenation and transformation from oxy-Hb to deoxy-Hb, the “pockets” with two GSH between two β-globin chains opens, allowing for the release of two GSH molecules from docking sites 1 and 2 (Fig. 5D, lower panel and movie M1). Superposition of oxy- and deoxy-Hb:GSH docking models and statistical analysis of hemoglobin residues involved in Hb:GSH complex formation are presented in Supplementary Fig. S6.

**Fig. 6:**
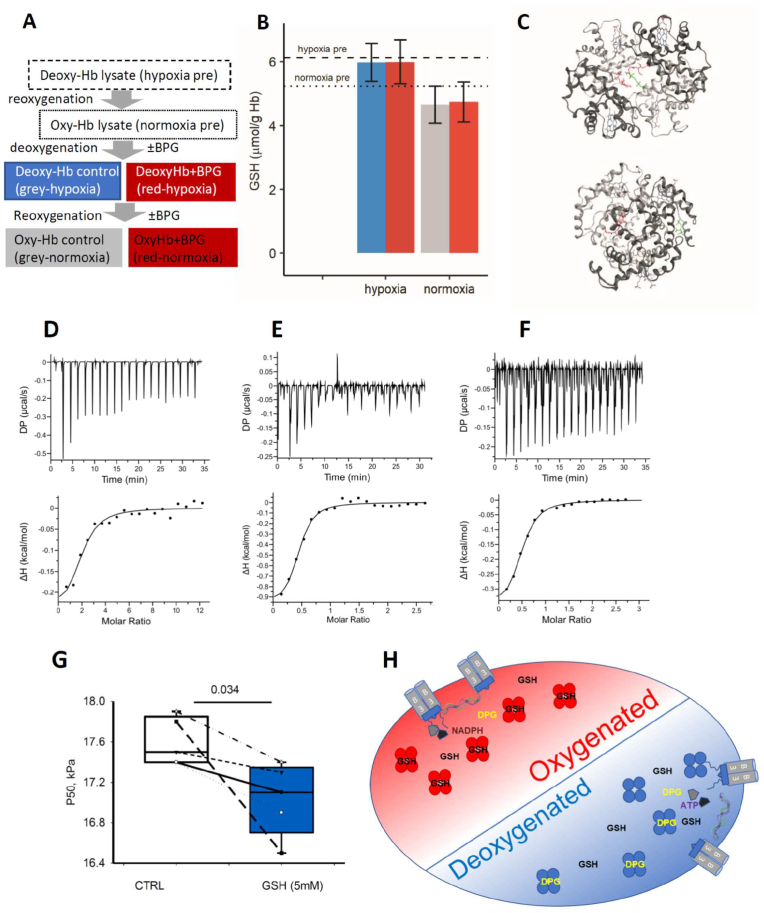
**The interplay between GSH and BPG binding to Hb and regulation of O**_**2**_**affinity of Hb.** The experimental design to assess the possible competition between BPG and GSH for binding to Hb (A) and the changes in free GSH in hemolysates that were produced by deoxygenation in pure N_2_, reoxygenation with air, supplementation of 2 mM BPG followed up by repeated deoxygenation (B). Dashed lines stand for the basal GSH levels in hypoxic and normoxic hemolysates. Red and blue bars stand for the free GSH levels in hypoxic BPG-treated and control hemolysates respectively. Grey and red bars show free GSH levels in reoxygenated control and BPG-treated hemolysates. N = 3. (C) Models showing interactions of GSH with BPG-Hb complex (front and side views). Alpha chains are shown in light grey, and β chains are in dark grey. GSH molecules are highlighted in red, and BPG molecules are in green. (D) ITC titration curve (upper panel) and binding isotherm (lower panel) for GSH binding to deoxy-Hb:BPG complex (E) interaction of BPG with deoxy-Hb in the absence or (F) in the presence of GSH at 25 °C. Thermodynamic parameters of these interactions determined by isothermal titration calorimetry are shown in Table S3 (G)P50 values obtained for hemolysates in the absence or presence of 5 mM GSH measured by Hemox analyser. (H) Schematic representation of a possible mechanism of changes in docking partners for Hb in RBCs during deoxygenation-reoxygenation (for details, see the text). *BPG: 2,3-bisphosphoglycerate, P50: the oxygen tension when hemoglobin is 50% saturated with oxygen. ΔH: the changes in enthalpy, -TΔS: the changes in entropy, and ΔG: the changes in Gibbs energy*. (For interpretation of the references to colour in this figure legend, the reader is referred to the Web version of this article.)

The central cavity of Hb harbors a binding site for 2,3-bisphosphoglycerate (BPG) which binds at the β-β globin interface. A competition experiment binding of GSH by oxygenated Hb and its release while Hb gets deoxygenated was assessed in the presence or absence of 2 mM BPG. The scheme of the experiment is presented in Fig. 6A and the outcome summarized in Fig. 6B. As follows from Fig. 6B, the presence of BPG does not interfere with O_2_-dependent binding or release of GSH from Hb.

*In silico* modelling supports these findings, predicting no interfering between the BPG and GSH binding sites (Fig. 6C). Docking of GSH to the complex deoxy-Hb-BPG is not disturbed and binding sites for BPG and GSH do not overlay spatially.

In-depth investigation of the binding constants (*K*_d_) and stoichiometry of GSH binding to complex deoxy-Hb:BPG was perfomed using ITC. The *K*_d_ values for GSH interaction with deoxygenated Hb were insensitive to the presence of BPG (Fig. 5, Fig. 6D, Table S3) revealing the accessibility of the GSH binding sites to GSH in deoxy-Hb:BPG complex. Based on the ITC data and on the modelling predictions, two out of four GSH molecules occupying the sites 1 and 2 (Fig. 5D) leave the Hb cavity during the transition from oxy-Hb to deoxy-Hb. Some of the amino acids forming BPG binding site in deoxy-Hb were predicted to be a part of the GSH binding site 1(Table S2). However, site 1 is not existing in deoxy-Hb conformation and, thus, no competition exists between GSH and BPG for binding to Hb. Docking of GSH to the sites 3 and 4 in deoxy-Hb did not interfere with BPG-deoxy-Hb complex formation (Fig. 5, Fig. 6C,E,F, Table S3) but caused a modest reduction in the enthalpy component and an increase in the entropy contribution to BPG binding to the central cavity of deoxy-Hb (Table S3). This indicates a decrease in the contribution of Van-der-Waals and hydrogen interactions and an increase in the contribution of hydrophobic interactions to the BPG-deoxy-Hb complex formation. Taken together, these findings indicate that the central cavity of oxy-Hb harbors the GSH binding site. The latter ceases to exist upon oxy-to-deoxy-Hb transition, while a new binding site for BPD is formed within the central cavity of deoxy-Hb.

Finally, the impact of GSH on O_2_ dissociation from Hb was assessed in RBC lysates. The addition of 5 mM of GSH to the lysates resulted in a small but consistent reduction in partial pressure at which SO_2_ reaches 50% (P_50_), indicating an increase in Hb O_2_ affinity (Fig. 6G).

## 4 Discussion

This study revealed a new function for Hb as a regulator of intracellular free GSH levels making them O_2_-sensitive. Our data indicated that the transformation of Hb from oxy-to deoxy-state results in dissociation of two out of four non-covalently bound GSH molecules from one Hb tetramer. Non-covalent interaction between Hb and GSH does not interfere with binding of BPG to deoxy-Hb but impacts the thermodynamics of BPG interaction within the central cavity of deoxyHb (Table S3). These findings are in line with several recent studies implying that the central cavity of Hb is a multi-ligand docking site for several molecules. Those ligands include organic phosphates, NAD(P)H [29], and, as we were able to show, GSH. Binding sites for all these negatively charged allosteric modulators share some common amino acids as a part of their docking interface. (Table S2). For example, Val1 of the β-chain is a common binding partner for Cl^−^ [30], BPG [31], and GSH (site 1, Table S2) and Lys82 for BPG [31,32] and GSH (site1, Table S2) binding. The probability for a particular ligand to bind to the central cavity of Hb depends on pH, temperature, ligand's concentration and affinity, Hb oxygenation, and the presence of competing ligands [32].

Cytosolic Hb concentration (5.4 mM) is comparable with that of GSH (1.2–3.5 mM) and BPG (2–7 mM) both depending on the severity of hypoxia [[33], [34], [35]] whereas the concentrations of NADH (10^−4^ mM) and NADPH (10^−2^ mM) are lower and comparable with GSSG levels [36]. Under conditions of chronic O_2_ deprivation such as HA exposure, the concentration of BPG rises [35] increasing the probability for BPG to bind to Hb even in an oxygenated state. Docking of BPG to the central cavity of Hb tetramer would compromise O_2_ loading in the lungs, even though its affinity to oxy-Hb is low (*K*_d_ = 4.75 mM for oxy-Hb vs 0.1 mM for deoxy-Hb [37]). As follows from our findings (Fig. 5C and 6, Table S3), BPG binding to deoxy-Hb is not compromised by GSH and *vice versa*, and the release of GSH from Hb during deoxygenation is not altered by BPG. The increase in SO_2_ to 50% or more (Figs. 1G and 2A) will result in binding of GSH to sites 1 and 2 and interference with BPG binding to Hb, thus promoting O_2_ binding to Hb in hypoxic lungs (Fig. 6G,C&H). When reaching the hypoxic periphery, GSH is released from the cavity, and BPG binds to the high affinity site (Fig. 6C&H). The deoxygenation threshold for release of GSH according to our measurements *in vitro* is about 50% SO_2_. This threshold is achieved in venous blood during endurance exercise when arterio-venous difference in SO_2_ increase from 28% to 79% [30]. Earlier on Rossi et al. have observed a difference in free GSH levels in blood samples that were simultaneously obtained from *vena cava* from abdominal aorta of the same rat and acidified with TCA solution [14]. Differences in between arterial SaO_2_ (∼98%) and mixed venous blood SvO_2_ (∼70%) in conscious humans or rats, and the SO_2_ threshold of 50% (Fig. 2A) may not always be reached. However, due to the differences in O_2_ consumption rate and in blood flow rate in different tissues, SvO_2_ values for resting humans have been reported as 69% for the brain, 37% for the heart, 66% for the liver/splanchnic bed, 92% for the kidneys, 71% for skeletal muscle, and 88% for skin [38]. Endurance exercise, suppression of respiration (when in anesthesia, as for the rats use in Ref. [14]), as well as environmental hypoxia will result in further decrease in SvO_2_ reaching 50% or less, and GSH will be then released from the “pockets” within Hb as observed for rats and mice earlier on [14,15].

*De novo* production of GSH occurs at a cost of 2 ATP molecules per 1 GSH molecule, while reduction of GSSG to GSH required NADPH. Liberation of GSH from deoxy-Hb may provide advanced antioxidant defense against the free radical burst from hypoxic mitochondria [8,39] without extra ATP or NADPH expenditure. As anaerobic glycolysis in the only source of ATP (and NAD(P)H) for the organism that is suddenly challenged with the shortage of O_2_, decrease in glucose consumption by hypoxic RBCs is an advantage that may support survival during acute hypoxic periods [40]. Thus, instead of depletion of the GSH or bulk reduced thiols’ pools, their accumulation is observed in deoxygenated RBCs (Fig. 1D&G, 2A, 3A-D&G). Prolonged periods of systemic hypoxia, however, promote the development of uncompensated oxidative stress condition (e.g. Fig. 3G, [10,11,41]).

The findings of the present study revealed that the free cytosolic GSH levels are constantly changing while RBC are circulating between the lungs and the peripheral tissues. Thus, we cannot tell what the actual “concentration” of GSH in the cytosol is. Simple calculations may indicate the proportion between the GSH liberated from the non-covalently bound pool is actually observed when RBC undergo deoxygenation, if we consider that all four “pockets” of a single oxy-Hb tetramer are occupied by GSH. Let us assume that mean corpuscular Hb concentration is 335 g/L or 5.23 mM. As Hb makes most of dry weight, and intracellular water refers to the dry weight of RBC as 0.67:0.33 [42]. Thus, free GSH concentration in fully oxygenated RBCs translates from an average of 2.5 μmol/g Hb (Fig. 2A) to 1.23 mmol/L cell water. Decrease in hemoglobin oxygen saturation from 98 to ∼20% (Fig. 2A) resulted in an increase in free GSH up to ∼6 μmol/g Hb which translates to 2.96 mmol/L cell water. Maximal amount of GSH that could be released by 5.23 mM oxy-Hb makes up 10.5 mM (two GSH molecules per 1 Hb molecule). Thus, reduction in SO_2_ to 20% causes liberation of 1.73 mM of GSH, which makes 16.5% of the maximal GSH storage capacity.

Most of our measurements of GSH were performed using Ellman's reagent (DTNB). This method is rather robust and well suited for detection of GSH in blood samples [43,44], although ascorbate, oxidants and free Fe^2+^ may cause GSH-independent production of the 2-nitro-5-thiobenzoate (TNB) [45]. However, reduction of DTNB by ascorbate is substantially slower and does not contribute to our GSH detection protocol in which DTNB was applied 3 min prior to the detection of TNB. Furthermore, while measuring GSH, Ellman's assay detects a mixture of any small non-protein thiol-containing molecules (e.g. cysteine, or sulfide), in addition to GSH, which is clearly dominating (∼98% [44]) over the other sources of non-protein thiols present in low micromolar concentrations (e.g. Ref. [24] for free Cys). The use of NMR spectroscopy allowed us to verify our conclusions and confirm that GSH release indeed occurs in RBCs upon deoxygenation. Specificity of GSSG detection with Ellman's reagent is achieved by using glutathione reductase to reduce GSSG to GSH, which is then measured.

Further technique that we were using to assess the levels of protein and non-protein reduced thiols in hypoxic and normoxic RBCs, was fluorescence of the adducts of thiols with monobromobimane. This fluorescent dye interacts with dissociated thiolate anions, and, hence, the reaction is pH-dependent [46,47]. Deoxygenation of Hb results in binding of protons to it (Haldane effect [48]), and alkalinization of the cytosol, which may promote dissociation of H^+^ from thiol groups and increase the interaction between the MBBR and the thiol groups, particularly with GSH that has a pKa of 8.56 [49]. Thus, alternative techniques that do not rely on deprotonation of thiol group pH-independent had to be used to confirm the increase in reduced thiol levels in the deoxygenated RBCs.

Although we did not observe any O_2_-dependent changes in Hb-SSG levels in our study, this source of GSH is there in RBCs and most likely may be used under harsh conditions such as smoking and disease (chronic renal failure, iron deficiency anemia, hyperlipidemia, diabetes mellitus, Friedreich's ataxia, atherosclerosis). All these pathologies were reported to be associated with advanced S-glutathionylation of Hb [50,51]. Similar to non-covalent binding of GSH to Hb (Fig. 6G), its S-glutathionylation was shown to increase Hb O_2_ affinity [[52], [53], [54]].

The intracellular GSH levels in RBCs are in dynamic equilibrium with the other molecules forming “redox environment”. Those include NO and reactive oxygen and nitrogen species. We have briefly examined the impact of hypoxia on the levels of NO and reactive oxygen/nitrogen species using fluorescent dyes. The use of flow cytometry on RBCs stained with membrane-permeable ester of diaminofluorescein (DAF) gave us an opportunity to monitor the relative changes in N_2_O_3_ derived from NO in single cells [55]. Heterogeneity of RBC is well-recognized [56], and single-cell approaches are essential to observe the possible presence of “non-responders” and “hyper-responders” in RBC population challenged by hypoxia. The same is true for free radical production and its detection by DHR123. Among the disadvantages of fluorescent dyes, such as DAF, are the inability to obtain quantitative readouts, the sensitivity of the dye to the changes in pH and to oxidants. In case of RBCs, it was also not possible to apply a blocker of NO synthases to obtain the negative control as non-enzymatic production of NO from NO_2_^−^ that is catalyzed by deoxyhemoglobin [57]. As non-enzymatic production of NO is increased with deoxygenation, the enzymatic generation of NO by endothelial NO synthase present in RBCs [58] gets suppressed as O_2_ is used for it as a substrate. Furthermore, upon production NO interacts with GSH and protein thiols forming S-nitrosylated adducts [[59], [60], [61], [62]], and with hemes of hemoglobin [62] partially escaping its detection by DAF. Thus, the changes in fluorescence of DAF observed while comparing the hypoxic and normoxic RBCs (Fig. 3F) reflect the “tip of the iceberg” of NO production and trapping. Luckily for us, acidification below pH of 6.2 (that reduces that fluorescence of the DAF probe [55]) and extensive increase in ROS levels were not associated with the changes in RBC oxygenation (Fig. 3E) and could not interfere with the measurements.

This study urges further inspection of the physiological role of GSH pools non-covalently bound to Hb in health and disease. Detection of intraerythrocytic GSH levels should include its correction for Hb O_2_ saturation. Finally, we may expect that depletion of the Hb:GSH pool would reflect the severity of chronic uncompensated oxidative stress.

## Funding

Swiss National Science Foundation, #CRSII5_180234 (AB)

Swiss National Science Foundation, #320030E_180227 (AB and GM)

German Research Foundation (DFG) #MA 1503/31-1 (HM)

German Research Foundation (DFG) #KA 2268/5-1 (LK).

European Union's Horizon 2020 research and innovation programme under grant agreement No 860436 — EVIDENCE — H2020-MSCA-ITN-2019 (LK and AB), Forschungskredit of the University of Zurich, #FK-21-055 (SF),

Russian Science Foundation, grant #19-14-00374 (IP, 10.13039/100006138EM, PZ, AAA, YuP) (Fig. 3, Fig. 4, Fig. 5, 6C–F and Supplementary).

We would like to acknowledge the work of Dr Asya Makhro and Mr Nikolay Bogdanov for their assistance in measurements of bulk thiol levels and intracellular GSH and GSSG in blood samples from the HA study.

## Author contribution

Conceptualization: SF, AB, IP, AA, LK, HM, GM.

Methodology: SF, AB, IP, AA, YT.

Investigation: SF, IP, AA, EM, PZ, AB, YuP, JG, VM, YT.

Visualization: SF, AA, AB, YuP, IP.

Writing – original draft: SF, AB, IP, AA, EM, YuP, VM, AM.

Writing – review & editing: all the coauthors.

## Disclosures

The co-authors have nothing to disclose.

## Declaration of competing interest

The authors of the manuscript REDOX-D-22-00013 have no conflict of interests to declare.

## Data Availability

Data will be made available on request.
