# Supplementary material for: Hemoglobin is an oxygen-dependent glutathione buffer adapting the intracellular reduced glutathione levels to oxygen availability
Source: Redox Biol. 2022 Nov 16;58:102535. doi: 10.1016/j.redox.2022.102535 (PMC9679038; doi:10.1016/j.redox.2022.102535)
Supplement: Multimedia component 1 [file mmc1.docx]

Hemoglobin is an oxygen-dependent glutathione buffer adapting intracellular redox state to oxygen availability

Simone Fenk, Elizaveta Melnikova, Anastasia Anashkina, Yuri Poluektov, Pavel Zaripov, Vladimir Mitkevich, Yaroslav V. Tkachev, Lars Kaestner, Giampaolo Minetti, Heimo Mairbäurl, Jeroen Goede, Alexander A Makarov, Irina Yu. Petrushanko, Anna Bogdanova*

*Corresponding author. Email: annab@access.uzh.ch

**Supplementary Materials and Methods**

## Blood samples and study participants

The study at high altitude was approved by the ethics committees of the University of Heidelberg, Germany, (S-066/2018) and the University of Bern, Switzerland (2018-01766). Twelve young male subjects took part in the study and blood samples were collected at sea level and high altitude (see Fig. 1A and (Klein et al., 2021)).

Further nine venous blood samples of healthy donors of both genders were provided by the Clinical Laboratory of Cantonal Hospital Winterthur, Switzerland. These samples were used for calibration of blood analyzers (CO-oximetry included). In agreement with the Helsinki convention, healthy study subjects gave informed consent for blood donation. Li-heparin was used as anticoagulant.  Hemoglobin oxygen saturation SO_2_, hemoglobin (Hb) concentration, and methemoglobin was measured on a blood gas analyzer (ABL825 FLEX, Radiometer) immediately after opening of the vacutainer.

Deidentified study participants’ data that underly the reported results will be made available 3 months after the publication for a period of 5 years after the publication date at <https://zenodo.org/>.

## Free intracellular GSH and GSSG measurement

GSH measurements were performed immediately after opening of the vacutainers with blood samples, in parallel to CO-oximetry. Ellman's reagent was used to determine the levels of GSH and GSSG in the incubated blood samples as described elsewhere (Tietze, 1969;Bogdanova et al., 2003). Briefly, whole blood (*in vivo* study) or RBC suspension (*ex vivo* experiments) was diluted 5X in ice cold 5% Trichloroacetic acid (TCA) solution and deproteinized during a 30 min incubation on ice. TCA and low temperature provide optimal conditions for prevention of GSH oxidation (Rossi et al., 2002). Denatured Hb and other proteins were pelleted by centrifugation for 10 min at 4°C. The supernatant containing non-protein thiols was collected and pH in it was adjusted to neutral with saturated Tris-OH solution (Tris(hydroxymethyl)aminomethane, Biosolve, 200923). The samples were then used for GSH and GSSG detection. The 200 µl aliquot of supernatant was diluted 800 µl in GSH buffer (100mM Na_2_HPO_4_, 10mM Na_2_EDTA, Sigma). For the measurement of total GSH (GSH and GSSG), glutathione reductase (10µg/mL, Sigma G6004) and NADPH (β-Nicotinamide adenine dinucleotide 2′-phosphate reduced tetrasodium salt hydrate, Sigma N1630) at final concentration of 40 µM were added to the diluted samples. Finally, DTNB (5,5′-Dithiobis(2-nitrobenzoic acid), Sigma D8130) at final concentration of 10 µM was added and the resulting samples incubated for 3 min at room temperature. The absorption was measured before (blank) and after incubation with DTNB at 412nm on a Lambda 25 UV/VIS spectrometer (Perkin Elmer) and blank values were subtracted. A calibration curve was produced with reduced GSH (Sigma, G4251) diluted in 0.1 N HCl and used to calculate the concentration of GSH in the sample that was then normalized to hemoglobin concentration (measured by Drabkin’s reagent (Hemoglobin transformations solution, LCN 043 Dr Lange, Hach Lange GmbH, Berlin, Germany). All chemicals used in this study were from Merck if not stated otherwise.

## Effect of hypoxic exposure on the intraerythrocytic GSH ex vivo

Oxygen-dependence of GSH levels in RBCs: RBC were washed in bicarbonate-containing plasma-like buffer (125mM NaCl, 25mM NaHCO_3_, 4mM KCl, 10mM glucose, 300µM L-Arginine, 300µM glutamic acid monosodium, 300µM glycine 10mM HEPES-imidazol (pH=7.40 at room temperature), all reagents from Sigma-Aldrich) and centrifuged at 1700xg for 5min for 3 times. The washed red blood cells were diluted in the bicarbonate-containing plasma-like buffer to about 30% hematocrit and transferred into the Eschweiler tonometers. Buthionine sulfoximine (BSO, Sigma B2515) at final concentration of 1mM was added to specific blood samples. First, samples were fully oxygenated for 30 min with a humidified gas mixture of 15% O_2_, 5% CO_2_ and 80% N_2_. Subsequently, the gas mixture was changed to 0.5% O_2_, 5% CO_2_ and 94.5% N_2_ and incubated for up to 2 hours. At different time points (0, 30, 60, 120min) an aliquot was collected and analysed for hemoglobin oxygen saturation using CO-oximeter function of the blood gas analyser ABL825 FLEX, (Radiometer). Reduced and oxidized glutathione (GSH and GSSG) levels were detected in the samples by Ellman’s reagent.

The impact of NEM on GSH release under hypoxic conditions: RBC were washed three times in the plasma-like buffer and spun at 1700 x g for 5min. The resulting RBC pellet was resuspended in plasma-like buffer to a hematocrit of 30%. The alkylating agent NEM (20mM) was used to derivatize reduced thiol groups and incubated for 20 min at room temperature and subsequently triple-washed in plasma-like buffer and resuspended to a hematocrit of about 30%. The same procedure was performed for the control sample without the addition of NEM. Subsequently, the samples were placed into the Eschweiler tonometers and equilibrated with the air (21% O_2_) or pure N_2_ as gas phase at 37°C until RBCs were fully oxygenated or deoxygenated. Glutathione levels were detected by a colorimetric assay (Ellman's reagent), and hemoglobin SO_2_ controlled using ABL825 FLEX blood gas analyser (Radiometer).

GSH levels in hemolysates treated with BPG: Washed RBC were resuspended in plasma-like buffer to a hematocrit of 20%. The cells were destroyed by snap-freezing in liquid N_2_. Two 4 ml-samples of the resulting lysates were re-oxygenated in two tonometers for 20 min. Aliquots of each oxygenated lysate (0.4 ml) were collected for measurements of SO_2_ and GSH at time zero. Thereafter the atmosphere in the tonometers was switched to pure N_2_ and lysates were deoxygenating for 15 min before the next aliquot was collected for SO_2_ and GSH detection. Thereafter, one of the samples was supplemented with 2,3-Diphospho-D-glyceric acid penta(cyclohexylammonium) salt (BPG) at a final concentration of 2 mM, and deoxy-Hb in lysates was allowed to interact with BPG for 15 min before the SO_2_ and GSH measurements were repeated. Thereafter, the samples were reoxygenated for 20 min and the SO_2_ and GSH were detected in a final set of aliquots.

## Flow cytometry

Ex vivo deoxygenation experiments: RBC were washed twice with the bicarbonate-containing plasma-like buffer: 125 mM NaCl, 25mM NaHCO_3_, 4mM KCl, 0.75mM MgSO_4_ and 0.015mM ZnCl_2_, 10mM glucose, 100µM L-Arginine, 200µM glutamic acid monosodium, 200µM glycine, 0.2mM Alanine, 0.6mM Glutamine, 20mM HEPES-imidazole, pH 7.4 at RT. RBC were pelleted at 1500g during 10 min. Then RBC were diluted in the same medium to about 30-40% hematocrit, divided into two parts, and transferred into the hypoxic chamber (95% N_2_, 4.5% CO_2_, 1% O_2,_ 37^o^C) (Whitley H45 HEPA Hypoxystation) or into the cell culture incubator (20% O_2_, 5% CO_2_) at 37^o^C. Samples were incubated for 3 h, after that 1 µl RBC suspension was mixed with 99 µl of the bicarbonate-containing plasma-like medium and fluorescent probes were added to the samples to detect reduced thiols, reactive oxygen species, ROS or NO (Petrushanko et al., 2006;van Cromvoirt et al., 2021). Bulk intracellular reduced thiols were measured using monobromobimane (mBBr, 20 μM, E_em_/E_ex_=394/490 nm, ThermoFisher Scientific), ROS assessed using dihydrorhodamine 123 (DHR, 7.5 μM, E_em_/E_ex_=507/525 nm, ThermoFisher Scientific) and N_2_O_3_ as a marker of NO was measured using 4,5-Diaminofluorescein diacetate (DAF-FM DA, 5 μM, E_em_/E_ex_=495/515 nm, ThermoFisher Scientific). RBCs were loaded with the fluorescent probes for 30 min at 37°C in the same conditions (hypoxia or normoxia) in the darkness. Thereafter, RBC was diluted by buffer equilibrated under hypoxic or normoxic condition and fluorescence was recorded using flow cytometer BD LSR Fortessa Flow Cytometer.

Samples from HA study: Reduced bulk (non-protein and protein) thiols were detected by flow cytometry (Gallios, Beckman Coulter or LSRFortessa, Becton Dickinson). Whole blood (2uL) was added to the 1ml staining solution consisting of 10 µM mBBr, (ThermoFisher M1378) in Plasma-like buffer supplemented with 0.1% bovine serum albumin, (van Cromvoirt et al., 2021)). Cells were incubated for 1h in the darkness at room temperature. Thereafter, RBC fluorescence was assessed. Data analyses were performed using the Kaluza software (Beckman Coulter).

## Isothermal Titration Calorimetry (ITC)

Purified Hb solution was prepared as follows. Human Hb (Sigma H7379 lot #SLCJ0443) was dissolved in 50 mM K-phosphate buffer (50 mM KCl, 34.8 mМ K_2_HPO_4_, 15.2 mM KH_2_PO_4_, 2 mM MgCl_2_, pH 7.4) at concentration of 0.1 mM. As the purified protein was in metHb state, reduction was performed with 5 mM (from the 1M stock in H_2_O) sodium dithionite (Dia-M). After 10 min of incubation, 500 µL of 0.1 mM reduced Hb was passed through the gel-filtration column (PD MiniTrap G-25; GE Healthcare). Elution was performed using 600 µL of 50 mM K-phosphate buffer. The column was preliminarily equilibrated with 50 mM K-phosphate buffer. GSH stock solution (40 mM) was prepared on the same buffer and used at a final concentration of 4 mM for the ITC experiment. Stock solution of BPG (100 mM) was prepared on 50 mM K-phosphate buffer and used at the concentration of 1 mM. Hb:GSH complex was prepared by mixing of 670 µM GSH to 75 uM Deoxy-Hb. The following sets of experiments were carried out: (i) titration of Oxy- or Deoxy-Hb with GSH, (ii) titration of Deoxy-Hb with BPG, (iii) titration of GSH-DeoxyHb complex with BPG, (iv) titration of DeoxyHb with BPG with the following titration with GSH.

Experiments were performed in the normoxic atmosphere of 21% O_2_ (air), or under hypoxic conditions (1% O_2_). To achieve hypoxia the ITC system was placed into a hypoxic chamber (Whitley H45 HEPA Hypoxystation) to reach stable deoxygenating conditions (99% N_2_, and 1% O_2_) (Fig. S1). Prior to the experiment under hypoxic conditions all the buffers and the column were equilibrated with the atmosphere of hypoxic chamber overnight. Stock solutions of GSH, Hb or sodium dithionite Na_2_S_2_O_4_ were prepared by dissolving weight samples in hypoxic buffer in the Hypoxystation glovebox. All manipulations with Hb during its preparation were also performed in hypoxic atmosphere.

The thermodynamic parameters for the GSH and BPG binding to Hb were determined using a MicroCal iTC200 instrument (GE Healthcare) and PEAQ-ITC (Malvern Pananlytical), as described elsewhere (23). Experiments were carried out at 25 °C in a 50 mM K-phosphate buffer. Aliquots of the ligand (GSH, 2.0 µl, 4 mM) were injected into the cell containing 70-100 µM Hb to obtain a complete binding isotherm. Aliquots of the BPG (2.0 µl, 1 mM) were injected into the measurement cell containing 70-100 µM deoxyHb to reach a complete binding isotherm. To detect the effective heat of binding, the heat of dilution was subtracted from the heat of the reaction. The resulting titration curves were fitted using the MicroCal Origin and Malvern software, assuming one set of binding sites. Equilibrium association (Ka) and dissociation (Kd) constants and the enthalpy change (∆H) were determined, and the changes in Gibbs energy (∆G), and the entropy change (∆S) were calculated from equation ∆G= - RT lnKa= ∆H-T∆S.

## Hemoglobin oxygen affinity measurement

Hb oxygen affinity was measured using Hemox analyzer (TCS Scientific, New Hope, PA). Blood samples were washed in PBS and resuspended to a hematocrit of about 30%. Then, the cells were lysed by freezing in liquid nitrogen.

Two buffers were prepared and adjusted to pH of 7.2. The first buffer was PBS that was used as a control and the second buffer was supplemented with 5mM GSH. Five ml of the buffer in the cuvette of the Hemox analyzer was supplemented with 20 µL hemolysate were added and the oxygen dissociation curve (ODC) was recorded at 37°C. The data was analyzed using the R software and the P50 were calculated where the oxygen saturation of Hb was 50%.

## Analysis of solvent-accessible surface area in Hb structures

Structures of oxygenated (5WOG; 5WOH and 1HHO), deoxygenated (1A3N; 2HHB and 2DN2) and of hemoglobin-BPG complex (1B86) were obtained from protein data bank (rcsb.org). Solvent accessible surface area (SASA) was calculated using Accessible Surface Area and Accessibility Calculation for Protein server (Center for informational biology, Ochanomizu University, http://cib.cf.ocha.ac.jp/bitool/ASA/) and GETAREA server (Sealy Center for Structural Biology, University of Texas Medical Branch, http://curie.utmb.edu/getarea.html) (43) using a spherical water probe with a fixed radius (1.4 Å). Analysis of the cysteine S-groups positioning was performed using Moe2015.10 software.

*Docking of GSH to Hb and Hb-BPG complex*

For docking of GSH to Hb structures of human oxy-Hb (1hho; 1lfq; 1lft; 1lfv; 1lfy; 1lfz; 1r1x; 5wog; 5woh; 6bb5) and deoxy-Hb (1a3n; 1b86; 1bij; 1bz0; 1bzz; 1c7b; 1c7c; 1g9v; 2dxm; 4hhb) were obtained from the protein data bank (rcsb.org). For docking of GSH to Hb-BPG complex we have used the structure of human Deoxy-Hb–BPG complex (1B86) from the protein data bank (rcsb.org). Structures were pre-processed using the AutoDockTools program (The Scripps Research Institute). Docking was performed using Autodock Vina. Moe2015.10 software (was applied for analysis of receptor-ligand interactions.

## Investigation of the possible impact of deoxygenation on the Hb S-glutathionylation state

*Ex vivo* hypoxia: RBC were washed in a plasma-like buffer and centrifuged at 1700xg for 5min for 3 times. Washed RBC were resuspended in plasma-like buffer to a hematocrit of 30% and incubated in the atmosphere of pure N_2_ or air in Eschweiler tonometers at 37°C. Aliquots of RBC suspensions were collected after 0, 2, 5, 10 and 20 min of incubation and analyzed for SO_2_ using CO-oximetry module of the ABL825 FLEX blood gas analyzer (Radiometer). S-glutathionylation of hemoglobin monomers was assessed by immunoblotting (for details see the following section). The aliquot of the suspension (40µL) was diluted in 200 µL non-denaturing lysis buffer (137mM NaCl, 1% Nonidet P-40 (Fluka 74385), 2mM EDTA, 20mM Tris-HCl pH 8) supplemented with a protease inhibitor (1:200) and N-ethylmaleimide (NEM, 25mM, Sigma 04259). The alkylating agent NEM was used to derivatize reduced thiol groups to avoid further oxidation. The samples were incubated on ice for 30 min and centrifuged at maximal speed for 10 min at 4°C. The supernatant was stored at -20°C until further analysis.

*In vivo* hypoxia: Blood samples collected from 12 study participants at sea level and high altitude (see **Fig. 1A** and (Klein et al., 2021) for more details). Plasma and buffy coat were removed, and the RBC pellet was snap-frozen in liquid nitrogen and stored at -80°C. Samples were thawed on ice and the alkylating agent N-ethylmaleimide (NEM, Sigma 04259**,** 25mM) in phosphate buffered saline (PBS; 137mM NaCl, 2.7mM KCl, 10mM Na_2_HPO_4_, 1.76mM KH_2_PO_4_) was added to derivatize reduced thiol groups to prevent artifactual oxidation in sample preparation.

To produce a negative control sample the lysate was treated with 100 mM dithiothreitol (DTT) in PBS. Thereafter 15µL of negative and control samples were mixed with 400uL PBS supplemented with 25mM NEM. Hemoglobin concentration in the resulting samples was detected using Drabkin’s solution (Drabkin, 1946). One volume of hemolysate was mixed with the five volumes Laemmli buffer (Laemmli, 1970) without β-mercaptoethanol and 10µg of protein were loaded on a 14% acrylamide gel, separated by SDS-PAGE and transferred to the nitrocellulose membrane. The membrane was fixed in 0.4% Paraformaldehyde in PBS for 30min. Subsequently, the membrane was blocked with 5% milk in Tris-buffered saline containing 0.1% Tween-20 (TBS-T; 150mM NaCl, 50mM TrisHCl, 0.1% Tween-20) for 30 min and incubated with the primary mouse Anti-Glutathione antibody for detection of S-glutathionylated proteins (ab19534, abcam 1:1000) in 5% BSA/TBS-T was incubated for 1h. Thereafter, the membrane was washed three times for 5 min with TBS-T and incubated with horseradish peroxidase (HRP)-conjugated secondary antibody in TBS-T for 30 min. The membrane was washed in TBS-T and developed using HRP substrate (32109, ThermoFisher), 10 sec). The GSH-associated signal was visualized on ChemiDocTM Imaging System (BioRad). Subsequently the membrane was washed 5 min in TBS-T and incubated with primary rabbit anti-human Recombinant Anti-Hemoglobin subunit beta/ba1 antibody (ab214049, Abcam 1:1000) in 5% BSA/TBS-T) for 1h with the following incubation with the corresponding anti-rabbit secondary antibody. Densitometry was performed using Image studio lite (LI-COR Biosciences) and the signal for GSH was normalized to that for the βHb monomer. Finally, the readouts obtained for different time points were normalized to the basal level of glutathionylation (time-zero for *ex vivo* experiments or the signal obtained for the blood samples before the exposure to HA).

## Statistics

Statistical analysis was performed using the R software (R version 3.6.1, R Core Team (2019). R: A language and environment for statistical computing. R Foundation for Statistical Computing, Vienna, Austria. URL https://www.R-project.org/) and GraphPad Prism (GraphPad Software) . Shapiro-Wilk test was used for checking if the data were normally distributed. Subsequently, a parametric (student’s t-test) or non-parametric test (Wilcoxon test) was used to compare the two experimental groups.  Specific details can be found in figure legends. Values are shown depending on their distribution either as means ± standard deviations or as boxplots with the minimum, the maximum, the sample median, and the first and third quartiles. To investigate the effects of the repeated measures in the in vivo study at high altitude, an analysis of variance (ANOVA) was performed with the study participant as a random factor. The differences between the 7 days were decomposed into four contrasts and tested on the level “day x study participant”. The first contrast tested whether the dependent variable was different at high altitude compared to sea level (* p value < 0.05, ** p value < 0.01, *** p value < 0.001). Then, the second contrast tested the difference between the two time points at high altitude (‡ p value < 0.05, ‡‡ p value < 0.01, ‡‡‡ p value < 0.001). Third, pre levels were compared to levels after return to sea level († p value < 0.05, †† p value < 0.01, ††† p value < 0.001) and the last contrast tested whether the days after return to sea level were different (¥).

**Figure S1.**


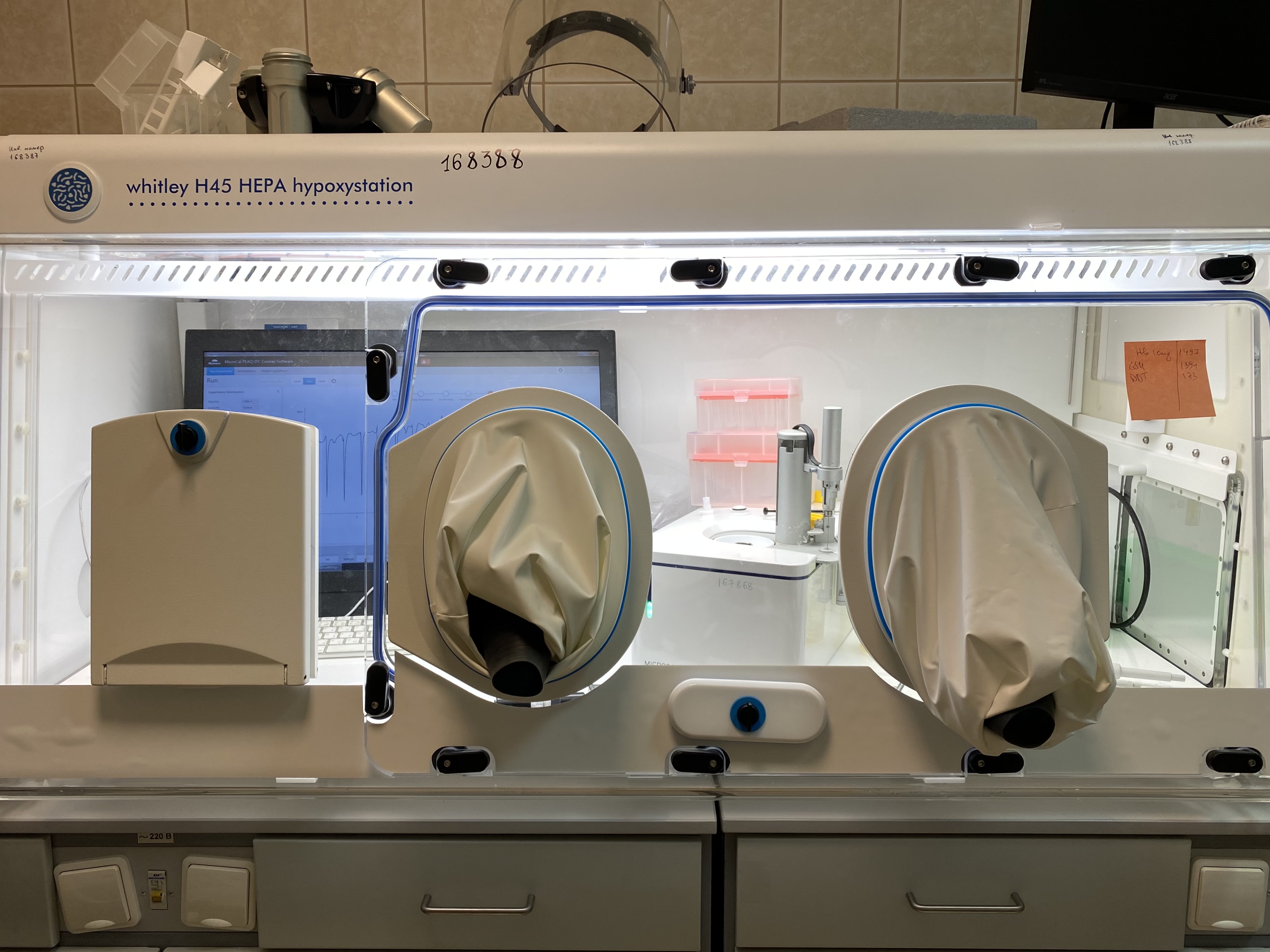


**Figure S1.** ITC system PEAQ-ITC (Malvern Pananlytical) is placed into the hypoxic glovebox chamber (Whitley H45 HEPA Hypoxystation).

**Figure S2A.** Donor 1 NMR GSH quantification data. Upper panels: region of ^1^H spectra series corresponding to Cys β-protons of reduced glutathione (two doublets), obtained by consecutive addition of standard GSH solution aliquots. Lower panels: linear least square fitting of ^1^H signal intensity against the amount of added standard.

| Hypoxic (1% O_2_) | Normoxic (20% O_2_) |
| --- | --- |
| 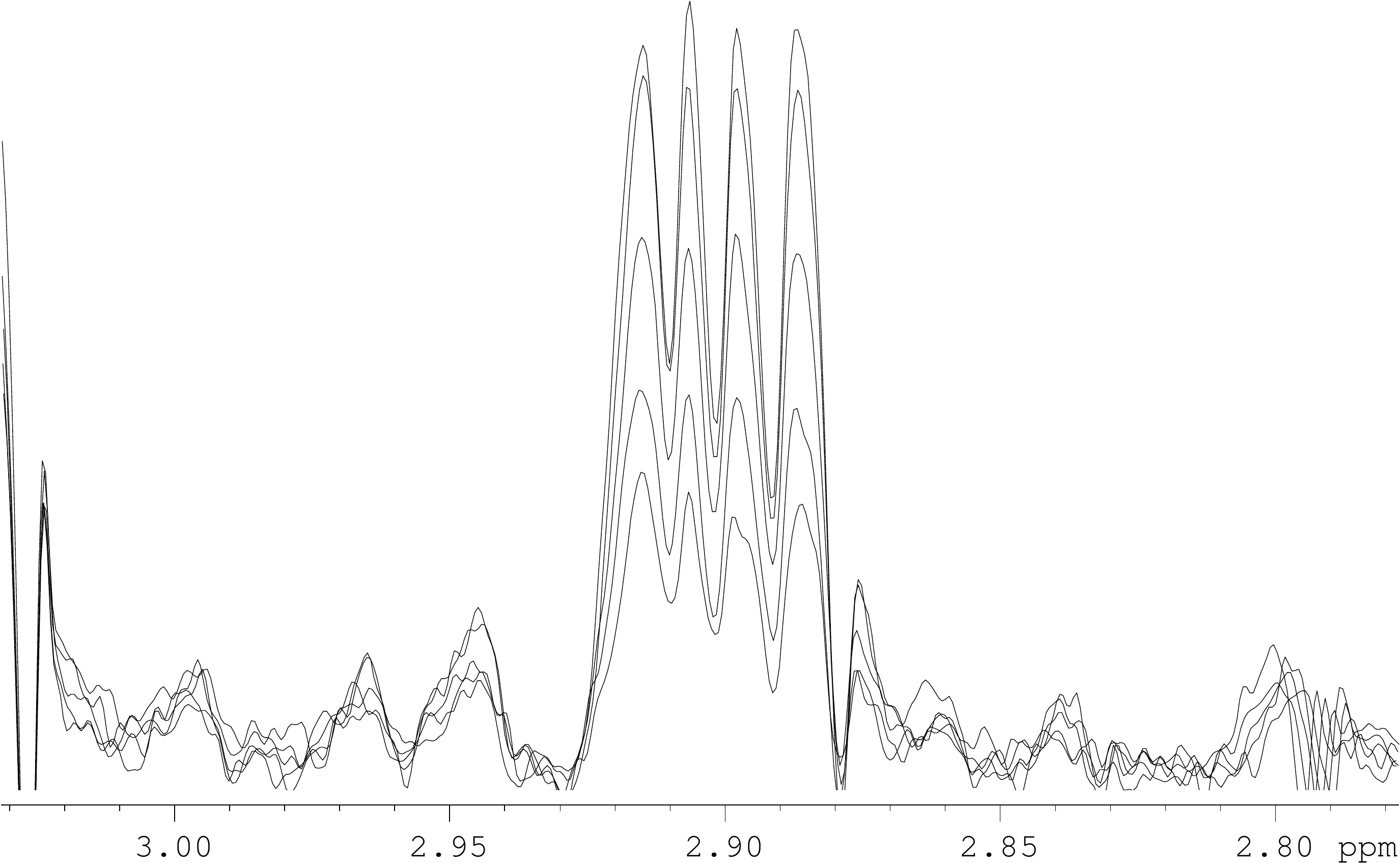 | 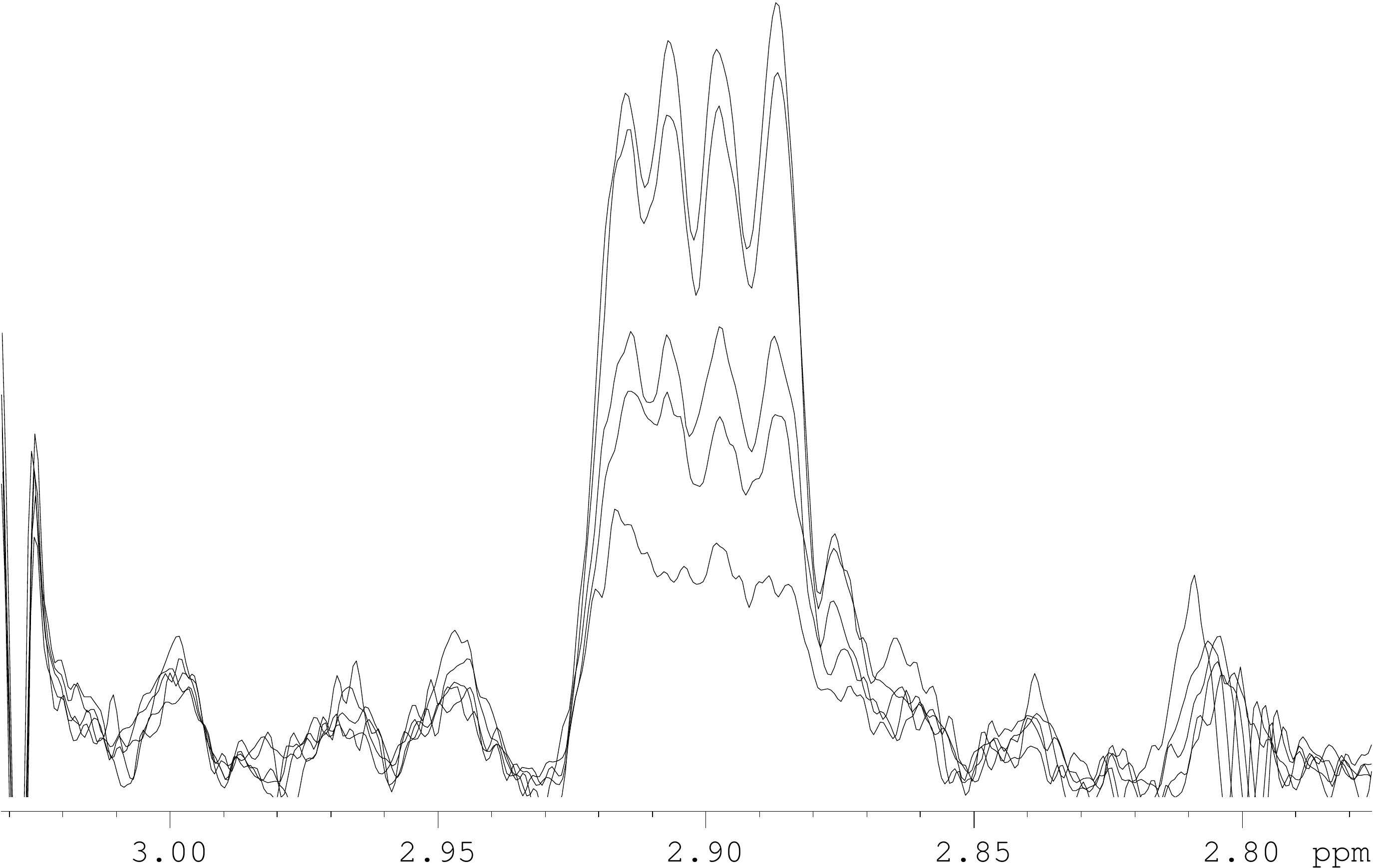 |
|  |  |

**Figure S2B.** Donor 2 NMR GSH quantification data. Upper panels: region of ^1^H spectra series corresponding to Cys β-protons of reduced glutathione (two doublets), obtained by consecutive addition of standard GSH solution aliquots. Lower panels: linear least square fitting of ^1^H signal intensity against the amount of added standard.

| Hypoxic (1% O_2_) | Normoxic (20% O_2_) |
| --- | --- |
| 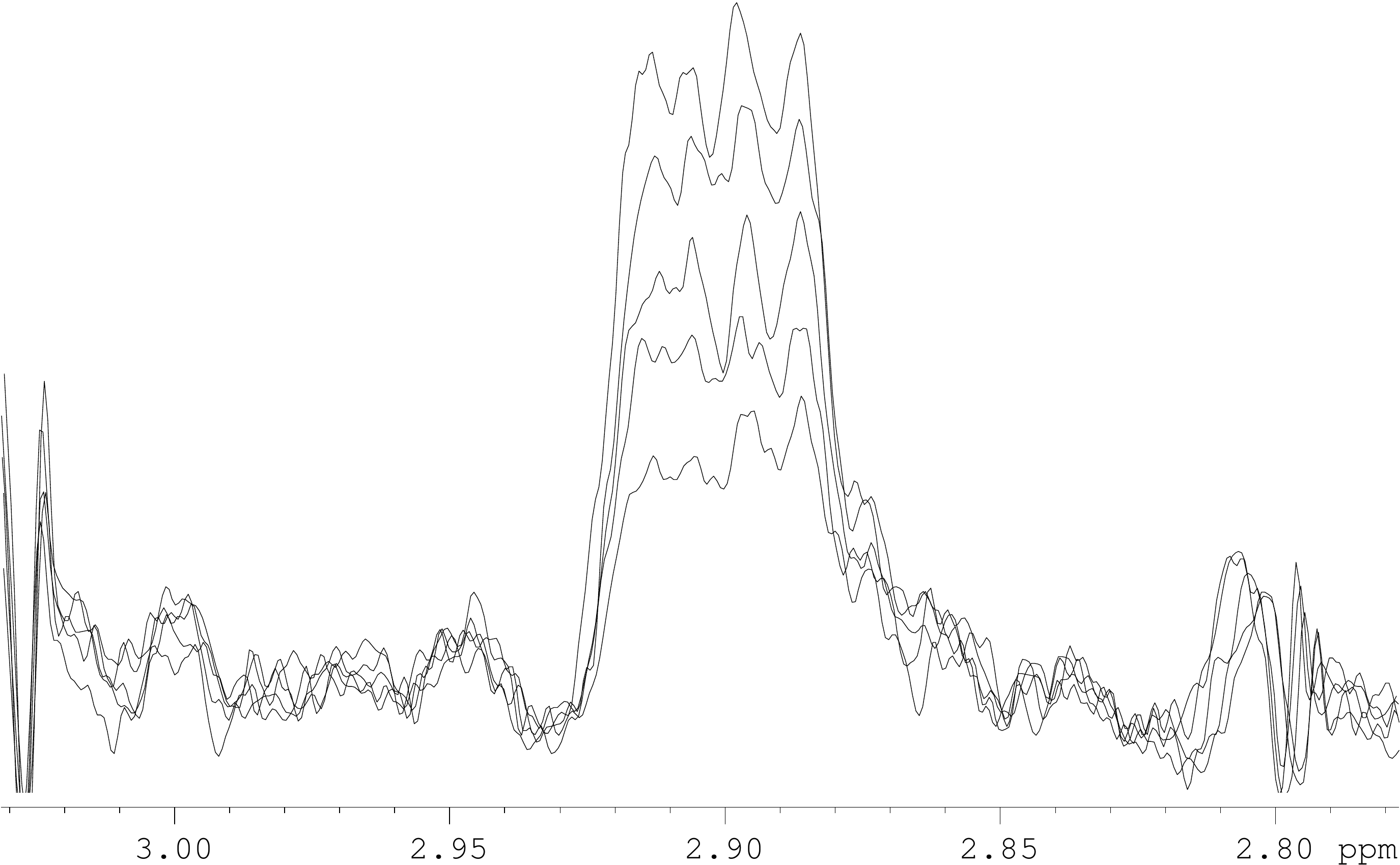 | 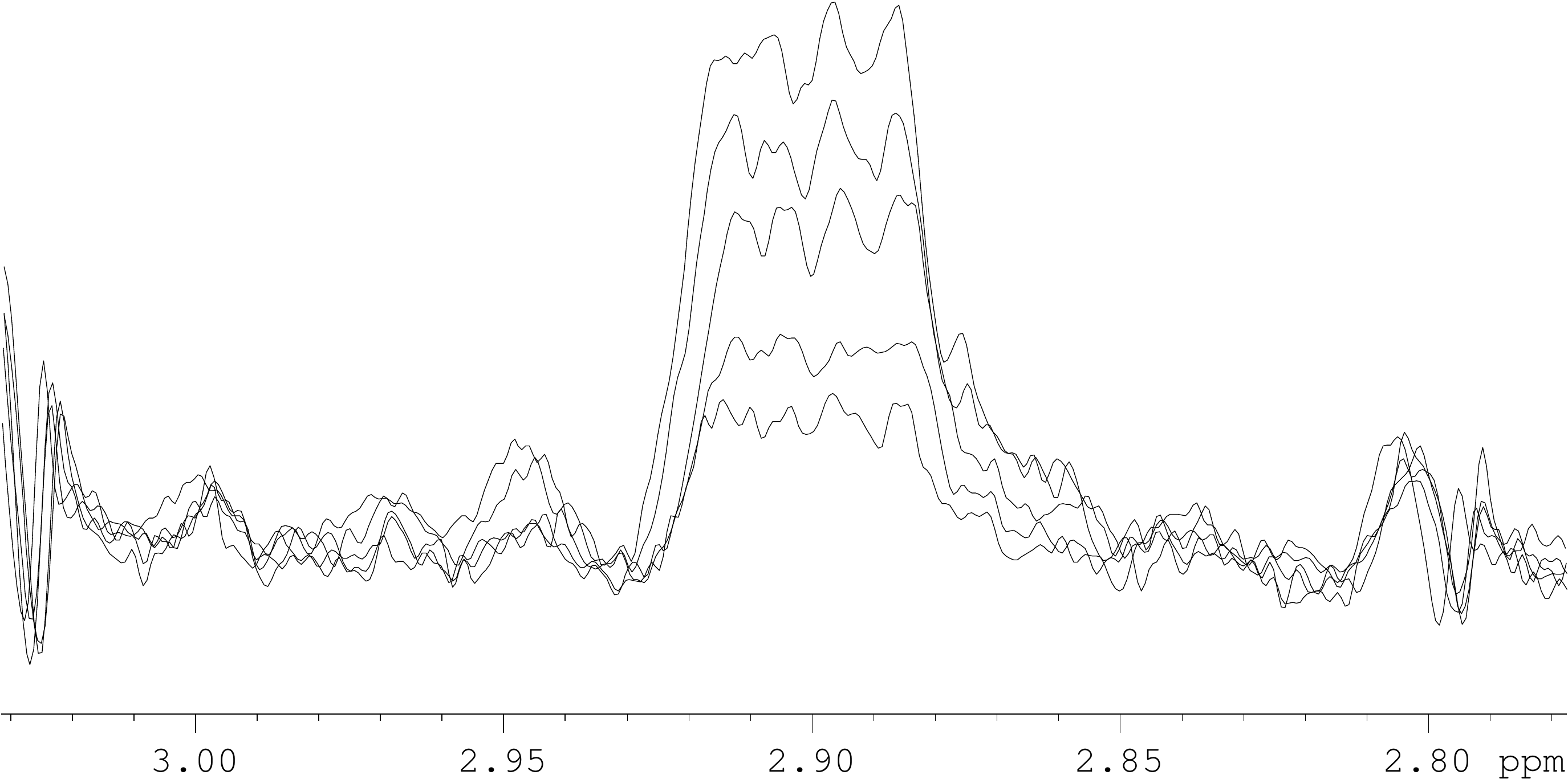 |
|  |  |

**Figure S2C.** Donor 3 NMR GSH quantification data. Upper panels: region of ^1^H spectra series corresponding to Cys β-protons of reduced glutathione (two doublets), obtained by consecutive addition of standard GSH solution aliquots. Lower panels: linear least square fitting of ^1^H signal intensity against the amount of added standard.

| Hypoxic (1% O_2_) | Normoxic (20% O_2_) |
| --- | --- |
| 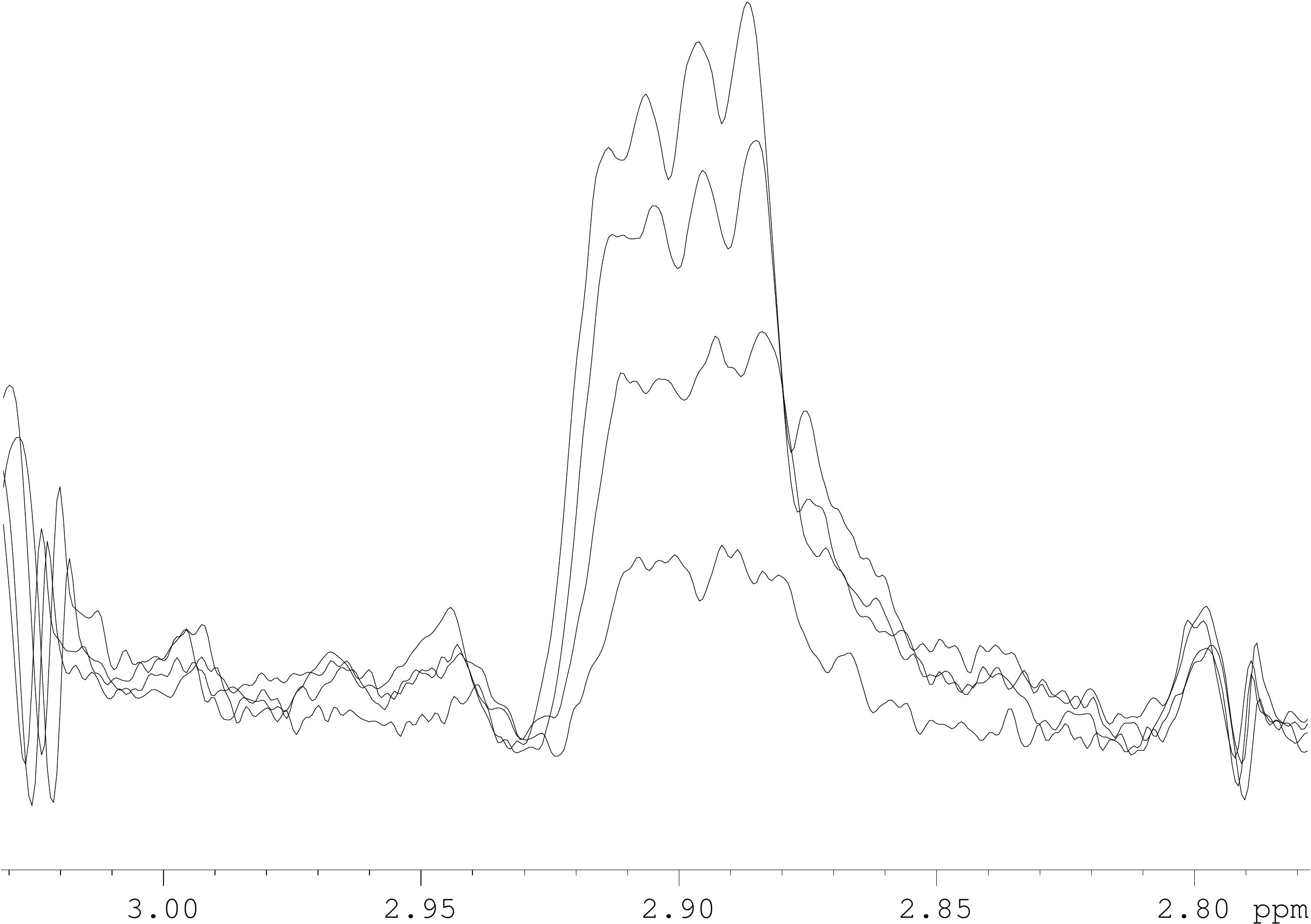 | 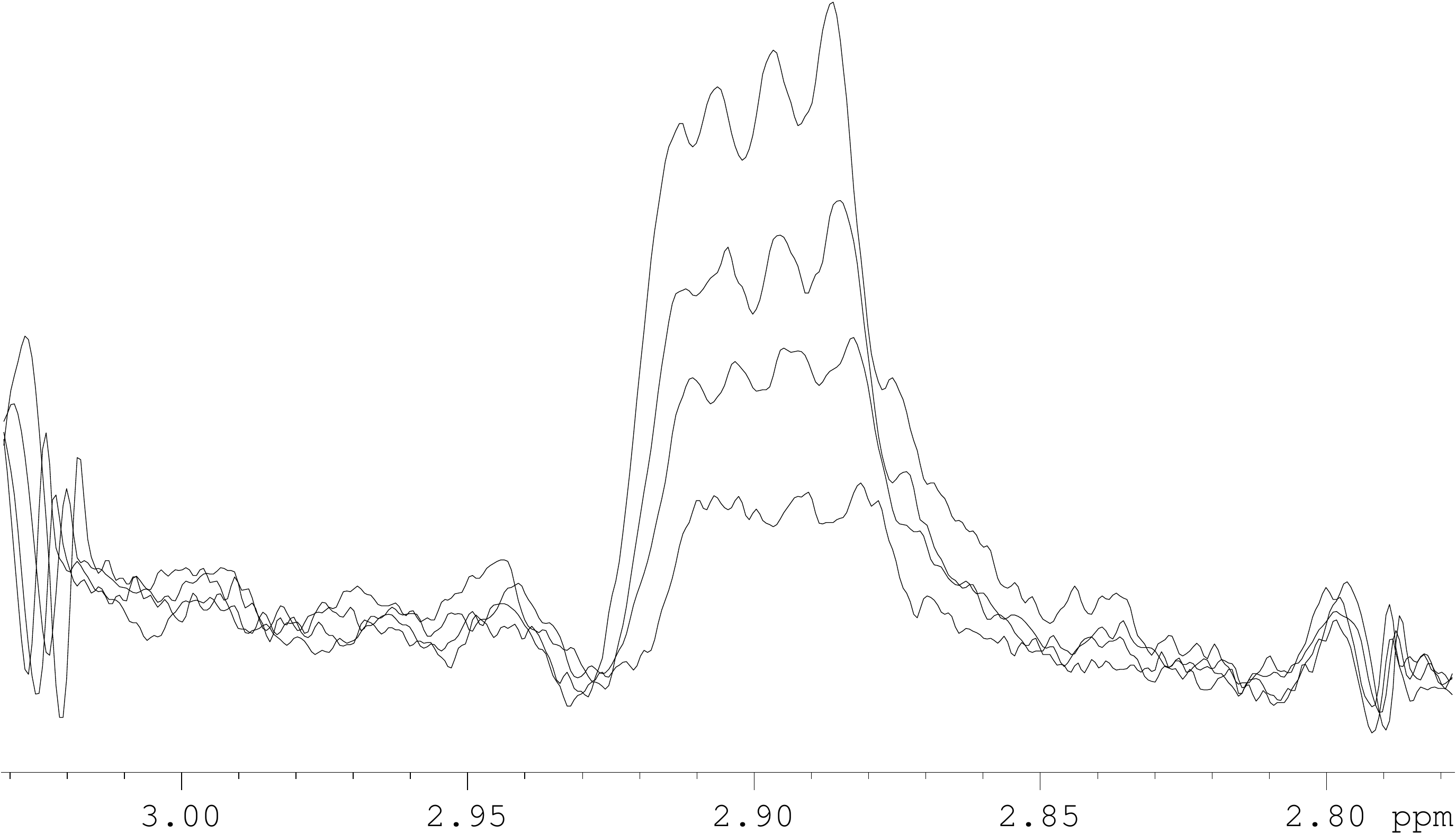 |
|  |  |

**Figure S2D.** Donor 4 NMR GSH quantification data. Upper panels: region of ^1^H spectra series corresponding to Cys β-protons of reduced glutathione (two doublets), obtained by consecutive addition of standard GSH solution aliquots. Lower panels: linear least square fitting of ^1^H signal intensity against the amount of added standard.

| Hypoxic (1% O_2_) | Normoxic (20% O_2_) |
| --- | --- |
| 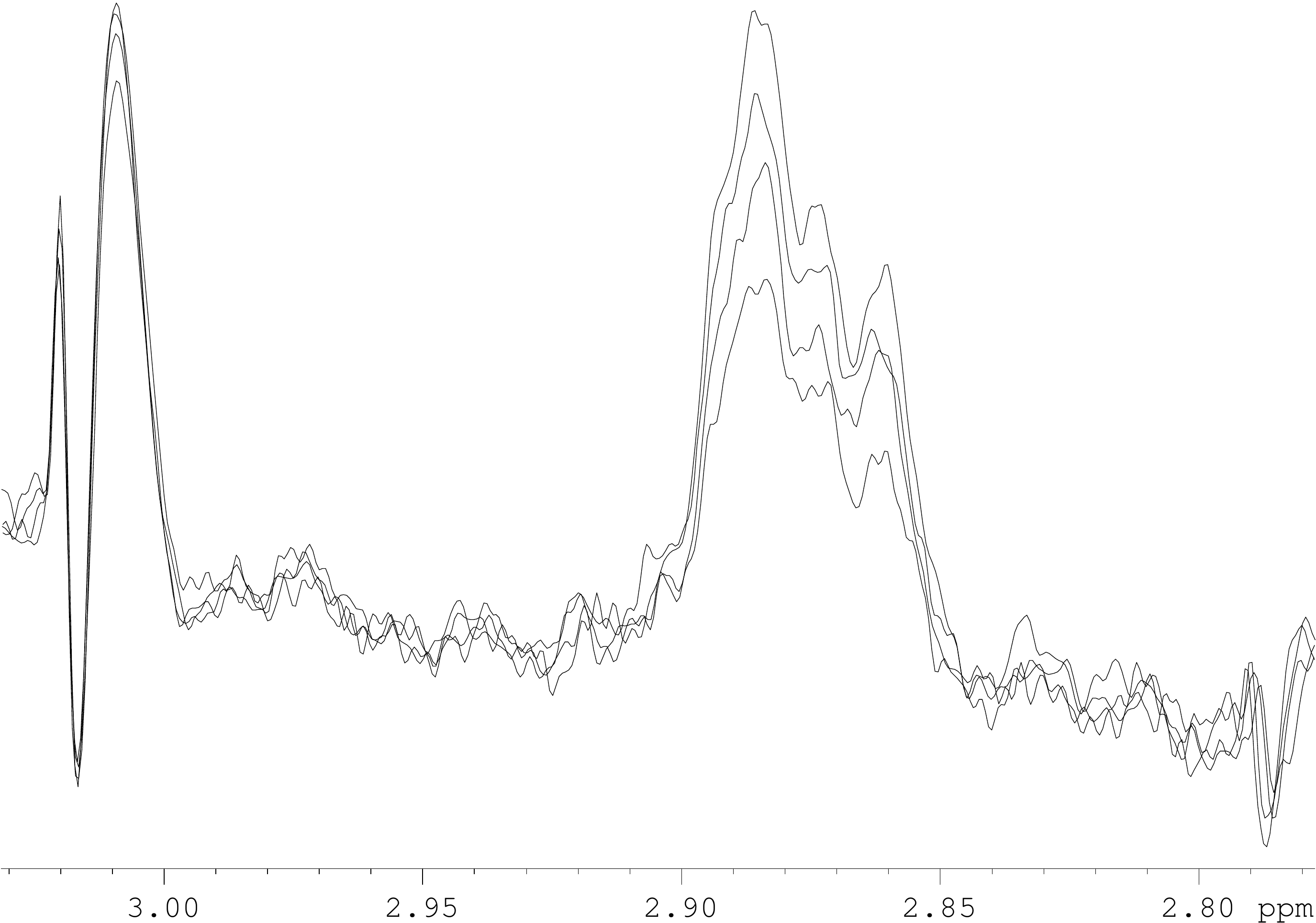 | 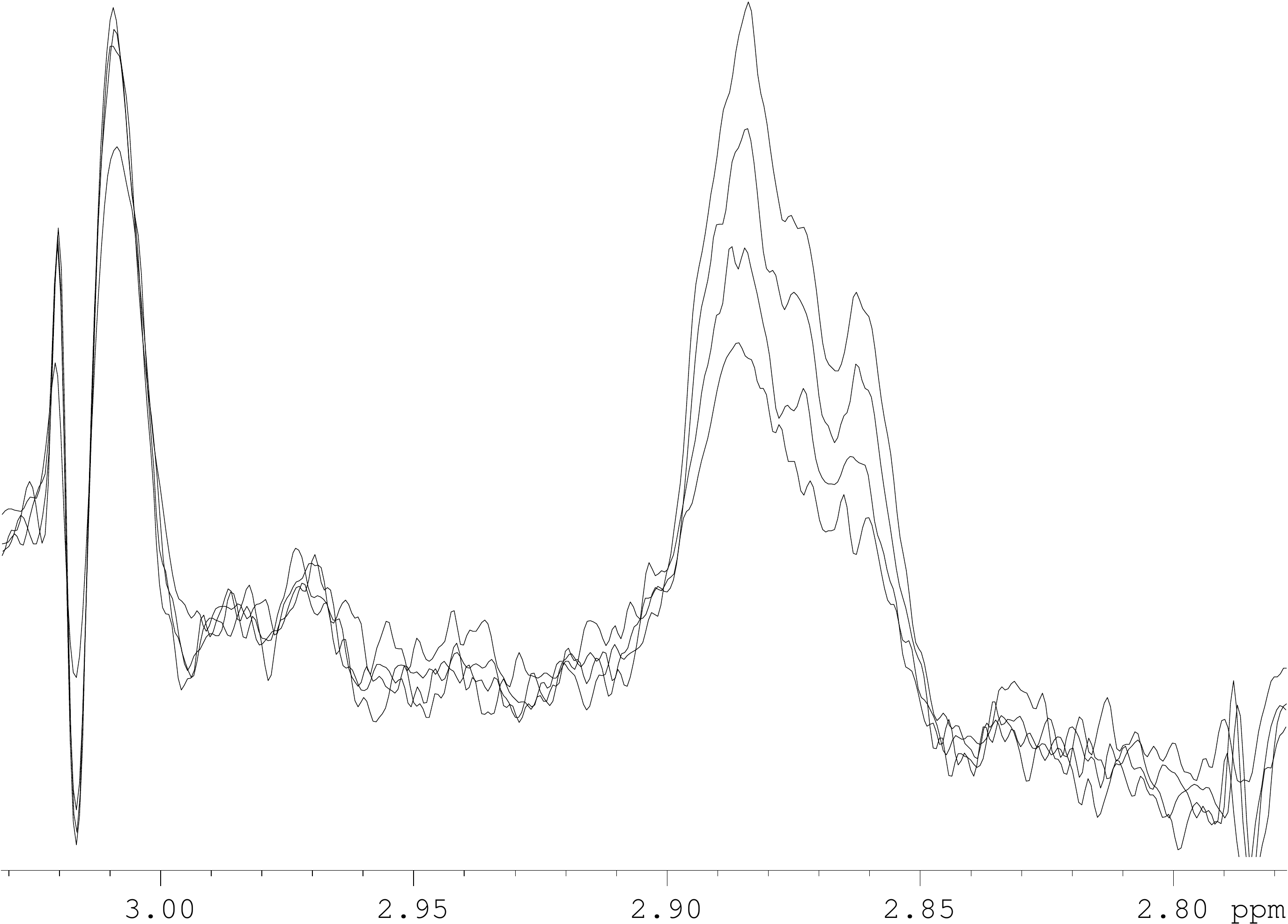 |
|  |  |

Figure S3.


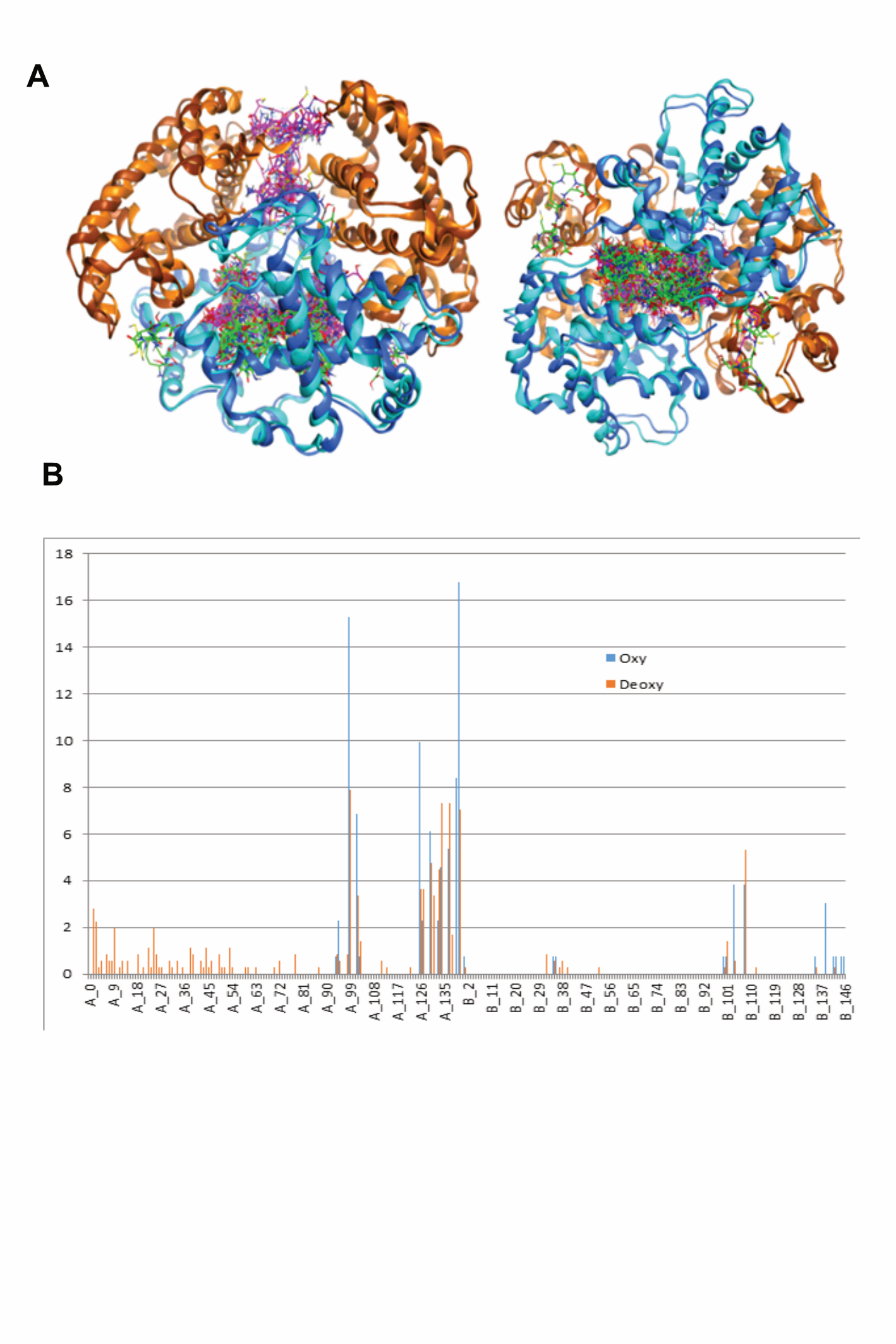


**Figure S6.** Docking of glutathione to the Hb molecule. A. Front and side views of superposition of oxy- and deoxy-Hb:GSH docking models. GSH molecules in pink are shown docked to the oxy-Hb. GSH docked to deoxy-Hb is shown in green. Alpha chains are shown in light blue (oxy-Hb) or dark blue (deoxy-Hb). Beta chains are in light brown (oxy-Hb) and brown (deoxy-Hb) B. The statistical analysis of hemoglobin residues involved in Hb:GSH complex formation by docking experiments. **Table S1**

**A**

| Oxyhemoglobin | | | | | |
| --- | --- | --- | --- | --- | --- |
| Cys residue | | | αCys104 | βCys93 | βCys112 |
| Structure reference (PDB id). | | 5WOG | 0 Å^2^ | 2.917 Å^2^ | 2.395 Å^2^ |
|  |  | 5WOH | 0 Å^2^ | 1.308 Å^2^ | 2.592 Å^2^ |
|  |  | 1HHO | 0 Å^2^ | 1.383 Å^2^ | 2.696 Å^2^ |
| AV | | | 0 | 1.869 | 2.561 |
| SD | | | 0 | 0.908 | 0.152 |
| Deoxyhemoglobin | | | | | |
| Cys residue | | | αCys104 | βCys93 | βCys112 |
| Structure reference (PDB id). | | 1A3N | 0 Å^2^ | 13.010 Å^2^ | 1.348 Å^2^ |
|  |  | 2HHB | 0 Å^2^ | 12.813 Å^2^ | 1.348 Å^2^ |
|  |  | 2DN2 | 0 Å^2^ | 13.285 Å^2^ | 2.489 Å^2^ |
| AV | | | 0 | 13.036 | 1.728 |
| SD | | | 0 | 0.237 | 0.658 |
| Deoxyhemoglobin in complex with DPG | | | | | |
| Cys residue | | | αCys104 | βCys93 | βCys112 |
| Structure reference (PDB id). | 1B86 | | 0 Å^2^ | 11.144 Å^2^ | 2.904 Å^2^ |

**B**

| Oxyhemoglobin | | | | | |
| --- | --- | --- | --- | --- | --- |
| Cys residue | | | αCys104 | βCys93 | βCys112 |
| Structure reference (PDB id). | | 5WOG | 0 Å^2^ | 2.72 Å^2^ | 2.56 Å^2^ |
|  |  | 5WOH | 0 Å^2^ | 1.57 Å^2^ | 2.60 Å^2^ |
|  |  | 1HHO | 0 Å^2^ | 1.23 Å^2^ | 2.59 Å^2^ |
| AV | | | 0 | 1.84 | 2.583 |
| SD | | | 0 | 0.78 | 0.02 |
| Deoxyhemoglobin | | | | | |
| Cys residue | | | αCys104 | βCys93 | βCys112 |
| Structure reference (PDB id). | | 1A3N | 0 Å^2^ | 11.08 Å^2^ | 1.46 Å^2^ |
|  |  | 2HHB | 0 Å^2^ | 10.97 Å^2^ | 1.71 Å^2^ |
|  |  | 2DN2 | 0 Å^2^ | 11.00 | 2.29 Å^2^ |
| AV | | | 0 | 11.01 | 1.82 |
| SD | | | 0 | 0.05 | 0.42 |
| Deoxyhemoglobin in complex with DPG | | | | | |
| Cys residue | | | αCys104 | βCys93 | βCys112 |
| Structure reference (PDB id). | 1B86 | | 0 Å^2^ | 8.98 Å^2^ | 2.73Å^2^ |

Table S1: Surface accessible to solvent in oxy-Hb and deoxy-Hb structures.

Using Accessible Surface Area and Accessibility Calculation for Protein server (A) and GETAREA server (B) we calculated solvent accessible surface area (SASA) which is characterized by the geometrical exposure (Å^2^) of amino acid residue atoms to the spherical water probe with a fixed radius (1.4 Å) rolling over a molecule and represents the free space around the single residue. βCys93 has the different SASA in oxygenated and deoxygenated hemoglobin structures. Presented data suggests that αCys104 is inaccessible both hemoglobin states, SASA of βCys112 remains comparable in all hemoglobin structures.

**Table S2.**

| Binding site IDs | Beta | Alpha | References |
| --- | --- | --- | --- |
| GSH Site 1  (only for Oxy-Hb) | **β_1_Val1**, **β_1_Leu81**, β_1_Lys82, β_1_Gly136, β_1_Asn139, **β_1_Ala140**, β_1_His143, **β_1_His146**  **β_2_Leu81**, β_2_Lys82, β_2_Gly136, β_2_Asn139, **β_2_Ala140**, β_2_His143, **β_2_His146** |  | The present study |
| GSH Site 2  (only for Oxy-Hb) | β_1_Glu101, β_1_Arg104, β_1_Asn139, β_1_Ala142, **β_1_His146**  β_2_Glu101, β_2_Phe103, β_2_Arg104, **β_2_Ala138**, β_2_Asn139, β_2_Ala142, **β_2_His146** | α_1_Val96 | The present study |
| GSH Site 3  (for Oxy- and Deoxy-Hb) | **β_2_Val34**, β_2_Tyr35, **β_2_Trp37**, β_2_Leu105, **β_2_Asn108** | α_1_Thr137 , α_1_Ser138, **α_1_Tyr140**,  **α_2_Val1**, **α_2_Pro95**, α_2_Val96, **α_2_Phe98**, α_2_Lys99, α_2_Ser102, **α_2_His103**, α_2_Leu106, **α_2_Asp126**, α_2_Lys127, α_2_Leu129, α_2_Ala130, α_2_Ser131, α_2_Ser133, α_2_Thr134, α_2_Thr137 | The present study |
| GSH Site 4  (for Oxy- and Deoxy-Hb) | **β_1_Val 34**, β_1_Tyr35, **β_1_Trp37**, β_1_Arg104, β_1_ Leu105, β_1_Leu106, **β_1_Asn108, β_1_Val109** | **α_1_Val1**, **α_1_Leu2, α_1_Pro95**, **α_1_Phe98**, α_1_Lys99, α_1_Ser102, **α_1_His103**, α_1_Leu106, **α_1_Asp126**, α_1_ Lys127, α_1_Leu129, α_1_Ala130, α_1_Ser131, α_1_Thr134, α_1_Thr137, α_1_Ser138  α_2_Thr137, **α_2_Tyr140** | The present study |
| BPG | **Val1, His2, Lys82, His143, Lys86** | S187, K202, H206, K209 | (Bunn and Jandl, 1970;Parashar et al., 2021) |
| NADH/ATP | **A82**, K90/ K61 | S187, K202, H206, K209 | (Parashar et al., 2021) |
| Cl^-^ | **Val1**, His2, **His82** | Ser131 | (Mairbaurl and Weber, 2012) |

**Table S2: GSH binding site on the hemoglobin structure.** Conservative residues are in bold.

**Table S3**

| **Sample** | **Ligand** | **N** | **Ка, М^-1^** | **Кd, µМ** | **ΔH,**  **kcal/mol** | **-TΔS, kcal/mol** | **ΔG, kcal/mol** |
| --- | --- | --- | --- | --- | --- | --- | --- |
| deoxyHb+BPG | GSH | 1.8 | 0.38×10^5^ | 26.5 | -0.26 | -5.99 | -6.25 |
| deoxyHb | BPG | 0.41 | 3.3×10^5^ | 3.0 | -1.00 | -6.48 | -7.48 |
| deoxyHb+GSH | BPG | 0.46 | 1.9×10^5^ | 5.2 | -0.38 | -6.83 | -7.21 |

**Table S3 Thermodynamic parameters of the BPG and GSH binding to Deoxy-Hb determined by isothermal titration calorimetry at 25 °C.**

К_а_ – equilibrium association constant, standard deviation did not exceed ±20%;

К_d_ –equilibrium dissociation constant; calculated as К_d_ = 1/К_а_;

ΔH – enthalpy variation; standard deviation did not exceed ±10%;

TΔS – entropy variation; standard deviation did not exceed ±10%;

ΔG – Gibbs energy; Calculated from the equation: ΔG=-RTlnK_a_;

**Movie M1**. Docking of GSH and BPG to the hemoglobin molecule in the oxy and deoxy state.

Oxy-Hb contains four bound GSH molecules inside the cavity, two of them at the β-β interface (sites 1 and 2) and two at the α-α chains interface (sites 3 and 4). In deoxy-Hb, two GSH molecules from the β-β subunits interface are released from the sites 1 and 2 allowing BPG to bind. Reoxygenation of Hbb is associated with release of BPG from its binding site and binding of two GSH molecules to the sites 1 and 2. The sites 3 and 4 remain occupied by GSH independent of the Hb conformation.
